# Consistency evaluation and performance optimization of deep learning-based auto-contouring for nasopharyngeal carcinoma

**DOI:** 10.1038/s41598-025-33567-6

**Published:** 2025-12-23

**Authors:** Linghui Yan, Yuhao Lin, Zirong Li, Liuling Wang, Xiaoting Lin, Jianming Ding, Zixuan Leng, Qichao Zhou, Chuanben Chen, Zhaodong Fei

**Affiliations:** 1 Department of Radiation Oncology, Clinical Oncology School of Fujian Medical University, Fujian Cancer Hospital, Fuma Road, Fuzhou, 350014 Fujian People’s Republic of China; 2 Department of Research Algorithms, Manteia Technologies Co., Ltd, 1903, B Tower, Zijin Plaza, No.1811 Huandao East Road, Xiamen, 361000 China

**Keywords:** Radiotherapy (RT), Nasopharyngeal carcinoma (NPC), Contour delineation, Deep learning (DL) models, Head and neck cancer, Radiotherapy

## Abstract

Contour delineation is crucial for ensuring the efficacy and side effects of radiotherapy (RT), but it inevitably involves inter-observer variability (IOV). Deep learning (DL) models have been used to assist in contour delineation, but further evaluation is needed to guide healthcare professionals in the judicious application of DL models. The contours of 22 anatomical structures and the gross tumor volume (GTV) for 30 patients with nasopharyngeal carcinoma were delineated using four DL models: AccuContour, RT-Viewer-contour, RT-Mind, and PVmed Contouring. The overall kappa values and generalized conformity indices of these contours were calculated to assess consistency. The Dice similarity coefficient (DSC), Relative Volume Difference (RVD), 95th percentile Hausdorff Distance (HD95), and Average Symmetric Surface Distance (ASSD) were calculated to evaluate the accuracy of the contours. Additionally, two innovative model frameworks were introduced to improve the fidelity and reliability of patient contour delineation. The consistency of the contours generated by the four DL models was poor for GTV, pituitary gland, temporal lobes, and temporomandibular joints. Marked differences were still observed between the contours generated by the models and the manual delineations by oncologists for the GTV, lens, optic nerves, pituitary glands temporomandibular joints, temporal lobes, and trachea. The model frameworks we proposed can effectively optimize the contours of GTV, brainstem, eyes, lens, and temporomandibular joints. The contours generated by DL models still have deficiencies in the application of nasopharyngeal carcinoma radiotherapy. To address this, two model frameworks were proposed to increase the robustness of automatic contouring.

**Supplementary Information:**

The online version contains supplementary material available at 10.1038/s41598-025-33567-6.

## Introduction

Radiotherapy (RT) stands as a pivotal treatment strategy for tumor, offering the promise of tumor control while simultaneously posing the risk of damaging adjacent healthy tissues. In the course of head and neck RT, there exists the risk of damaging over 20 critical organs at risk (OARs), which could inadvertently trigger a spectrum of adverse effects, including xerostomia, dysphagia, hypopituitarism, and other lower cranial neuropathies. Consequently, a paramount responsibility for radiation oncologists during the RT planning phase is the scrupulous assurance that the radiation doses delivered to these OARs remain confined to acceptable safety margins.

Contour delineation, which is paramount in influencing the therapeutic efficacy and side effect profile of RT, is recognized as an exceedingly time-intensive duty for radiation oncologists. It demands a high level of expertise and can extend up to 180 min for complex head and neck regions^1^. The process is susceptible to inter-observer variability (IOV), arising from discrepancies in professional knowledge, clinical experience, and selection of imaging modalities among practitioners. This IOV can persist insidiously, even when employing diverse imaging methods^2^ or adhering to guidelines^3^. Moreover, extant literature substantiates that IOV in the delineation of target volume could escalate the toxicity associated with RT and adversely impact patient survival^4^. Therefore, mitigating IOV and bolstering the uniformity in contour delineation present a formidable challenge for radiation oncologists.

Deep learning (DL) models have emerged as powerful tools with the potential to improve quality, standardization, and efficiency in RT workflows^5^. By leveraging automated software for contouring, the aim is to reduce the time spent on manual delineation, attenuate IOV, and improve the uniformity and accuracy of dose distributions^6,7^. A growing number of commercial DL-based auto-contouring systems have been introduced to assist radiation oncologists with head-and-neck target and OAR delineation in clinical practice or prospective evaluation studies, with early reports suggesting that they can reduce contouring time while maintaining clinically acceptable accuracy in selected settings^7–13^. However, most prior investigations have focused on the performance of a single auto-segmentation system—often in a research or pre-clinical setting—rather than on systematic comparison of multiple commercial tools within the same clinical scenario. Furthermore, even when a model performs well on average, its generalizability can be limited: small subsets of cases that fall outside the training data distribution may still yield suboptimal contours, underscoring the need for rigorous, ongoing validation before routine clinical use^7,8,14^. In addition to manual IOV among clinicians, differences in training data, network architectures, and post-processing strategies can introduce inter-model variability between distinct DL auto-contouring systems, complicating efforts to harmonize treatment protocols across centers^6^. While several studies have evaluated individual commercial or in-house DL models for head and neck auto-segmentation^7,8,10–13^, there is a relative paucity of work systematically assessing the consistency between multiple commercial systems and exploring methods to stabilize their combined output.

Against this background, the present study has three main aims. First, we seek to quantify the inter-model variability of four commercial DL auto-contouring systems for the gross tumour volume (GTV) and a broad set of OARs in nasopharyngeal carcinoma (NPC). Second, we aim to identify specific structures that require heightened clinical attention because they are particularly vulnerable to disagreement between models. Third, we propose and evaluate two post-processing frameworks—one based on an image-confidence algorithm and the other on a deep-learning fusion strategy—designed to enhance the robustness of automatic contours without degrading the baseline performance of the best individual model. Together, these contributions extend prior work by moving beyond single-model evaluation to a systematic analysis of inter-model variability and by introducing practical fusion-based approaches to improve the stability of commercial auto-contouring in NPC.

## Methods

### Dataset preparation

30 patients with previously untreated nasopharyngeal carcinoma who received radical RT at our hospital were included in this study. Planning computed tomography (CT) images were acquired on a Brilliance CT Big Bore scanner (Philips Healthcare, Cleveland, OH, USA) with a slice thickness of 3.0 mm, covering from the vertex to 2 cm below the clavicle. Each patient’s dataset comprised CT images and contour files generated by four distinct DL models. Contours on the planning CT images, serving as reference standards, were meticulously delineated on AccuContour software (Version 3.0, http://www.manteiatech.com/). The GTV and OARs were delineated on CT imaging by a single observer (clinical radiation therapist, 10 years of experience) under the guidance of an experienced senior radiation oncologist (20 years of experience) according to consensus^15^. Pretreatment contrast-enhanced magnetic resonance imaging (MRI) and/or diagnostic positron emission tomography–computed tomography (PET-CT), when available, were used as auxiliary reference imaging to assist GTV delineation on the planning CT; PET-CT was not used as the primary CT dataset for RT planning. The study was conducted in compliance with the Declaration of Helsinki, and the Ethics Committee of Fujian Cancer Hospital approved this research (Ethics Approval Number: YKT2020-011-01).

### Deep learning model preparation

Four comprehensively trained NPC delineation models were prepared, identified as follows: AccuContour (Manteia Medical Technologies Co., Ltd.), RT-Viewer-contour (Linkingmed Technology Co., Ltd.) (https://www.linkingmed.com/), RT-Mind (Medmind Co., Ltd.) (http://www.medicalmind.cn), and PVmed Contouring (Guangzhou Perception Vision Medical Technologies Co., Ltd.) (https://www.pvmedtech.com/). These models were applied to each patient case to generate contours for a specified list of Regions of Interest (ROI) pertinent to NPC. 22 organs that were common to all 4 models were identified: body, brainstem, esophagus, eyes, lens, mandible, optic nerves, pituitary glands, parotid glands, submandibular glands, spinal cord, temporal lobes, temporomandibular joints, thyroid gland, and trachea. It should be noted that GTV delineations were exclusively derived from the outputs of AccuContour and RT-Mind. According to information provided by the vendors, the commercial DL models evaluated in this study were developed independently by their respective companies using proprietary multicentre training datasets. The 30 nasopharyngeal carcinoma cases used for the comparative evaluation were retrospectively identified from our institutional database and, to the best of the authors’ knowledge, were not used for training, fine-tuning, or internal validation of these commercial models. Because the commercial training datasets are anonymized multicentre collections, a small inadvertent overlap cannot be completely excluded and is therefore acknowledged as a potential limitation.

### Consistency analysis

First, the “Computational Environment for Radiotherapy Research (CERR)”^16^ was used to calculate the volumes of each OARs and GTVs for each patient, as well as the minimum, maximum, mean, standard deviation, intersection, and union volumes per case. The latest version of CERR includes a consensus tool for more probabilistic and quantitative analyses, such as apparent consistency, kappa-corrected consistency, and “Simultaneous Truth and Performance Level Estimation (STAPLE)” based on probability, sensitivity, specificity, and the overall Kappa coefficient with its associated p-value (testing the null hypothesis κ = 0).

In studies assessing consensus on target volumes, the overall kappa value is frequently employed as a single metric for evaluating consistency. Based on the kappa value, the overall agreement is categorized as follows: 0 represents no agreement, 0-0.2 indicates slight agreement, 0.21–0.4 suggests fair agreement, 0.41–0.6 implies moderate agreement, 0.61–0.8 denotes substantial agreement, and values above 0.81 signify almost perfect agreement^17^.

The conformity index (CI) serves as an alternative conventional approach for assessing agreement. The generalized CI (CI-gen) introduced by Kouwenhoven et al.^18^. was employed, as this method is not limited by the number of observers. The specific calculation methods for CI-gen are provided in Supplementary Material (Supplement 1).

### Assessment of clinical feasibility

The clinically acceptable manual contours were selected as a substitute for the gold standard. The contours generated by four DL models were compared against the manual contours. And 4 commonly used contour metrics were selected to ensure the reliability of results. Specifically, these encompass the Dice similarity coefficient (DSC)^19^, Relative Volume Difference (RVD), the 95th percentile Hausdorff distance (HD95)^20^, and Average Symmetric Surface Distance (ASSD)^21–23^. The specific calculation methods for the four contour metrics were detailed in the supplementary material (Supplement1). All structures were transferred to the same CT image, and contour statistics were analyzed in AccuContour software.

### Model architecture

To provide potential improvements in treatment planning and outcomes for affected patients, this study aimed to enhance the robustness and precision of contour delineation by building upon established deep learning architectures. Two principal model frameworks were proposed, each tailored to harness the unique strengths of their respective computational paradigms.

Method A (confidence-based fusion) is based on the image confidence algorithm originally proposed by Yang et al.^24^, which assigns a confidence score to each voxel within a ROI using local image information. In this framework, the four commercial DL models first generate binary segmentation masks for each structure. The voxel-wise intersection and union of these masks are then used to construct a trimap: voxels inside the intersection are treated as definite foreground, voxels outside the union as definite background, and the remaining voxels as an uncertain region. This trimap is processed by a three-dimensional image confidence algorithm that computes a spatial correlation matrix in the feature space of the planning CT and assigns each uncertain voxel a confidence value between 0 and 1 based on its similarity to foreground and background voxels. For each ROI, a confidence threshold is selected on the training set to maximise the DSC, and this threshold is then applied to obtain the final segmentation. In this way, the inter-model variability is concentrated in the uncertain region and refined using the underlying image characteristics, while regions of strong agreement are preserved. This approach requires no additional network training.

Method B (deep learning fusion) extends the output of the image confidence algorithm by incorporating deep learning. While Method A effectively integrates multiple segmentation results to produce a confidence map, its reliance on empirically defined thresholds may introduce variability across patients. To address this, Method B takes the planning CT, a fused contour map summarizing the agreement pattern across the four DL models (obtained by summing and normalizing their binary masks), and the confidence map generated by Method A, and concatenates them as multi-channel inputs to a nnU-Net–based architecture. The network is trained with a combined Dice and cross-entropy loss, allowing it to learn systematic bias patterns in individual model outputs and to correct them using both multi-model contour information and local image features, while preserving the strengths of the best-performing models.

The two techniques (Method A and Method B) were described in detail in the supplementary material (Supplement1). The accuracy of proposed methods were evaluated with DSC. This metric provided insights into the spatial accuracy of model’s predictions by assessing the overlap between predicted segmentations and the ground truth. To fulfill the data quantity requirements of the model, the dataset was expanded by incorporating an additional 31 patients with previously untreated nasopharyngeal carcinoma. The resulting 61 cases were randomly split into a training set (*n* = 48) and an independent test set (*n* = 13).

### Statistical analysis

Continuous geometric evaluation metrics, including the DSC, RVD, HD95, and ASSD, were summarized as mean ± standard deviation for each ROI and model. Because the main aim of the study was to describe patterns of variability and robustness across multiple DL models rather than to establish pairwise statistical superiority for every individual structure, and because performing formal hypothesis tests for each combination of structure and metric would result in extensive multiple comparisons with substantially reduced power after correction and a large number of potentially misleading p-values, these measures were analyzed descriptively, focusing on the magnitude and clinical relevance of the observed differences. For the inter-model agreement analysis, the overall Kappa coefficient and its associated p-value were calculated using the Kappa statistics module of CERR under the null hypothesis that the agreement among contours is no better than chance (κ = 0), so that *p* < 0.001 indicates agreement significantly higher than random.

## Results

### Consistency analysis

**Fig. 1:**
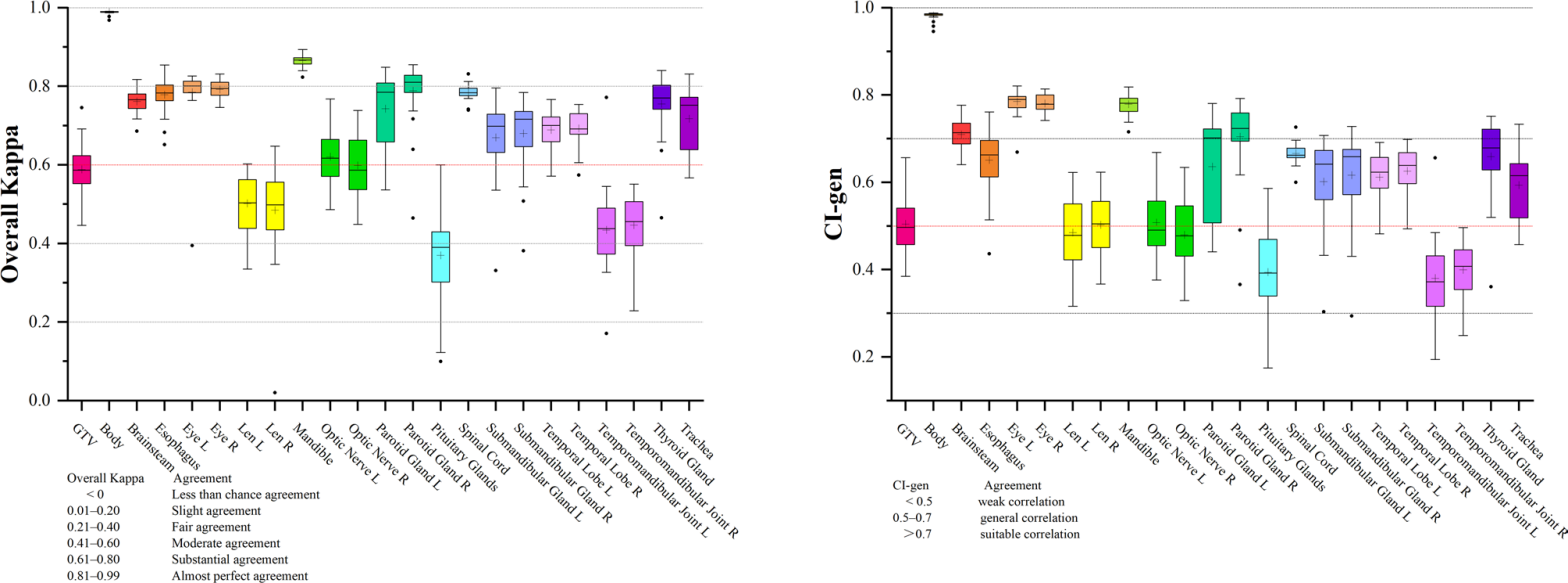
Box plots of overall kappa and CI-gen for GTV and OAR contours generated by 4 deep learning models in 30 nasopharyngeal carcinoma patients. ROI (Regions of Interest), GTV (Gross Tumor Volume).

This study first analyzed the GTV and OARs generated by four DL models in 30 cases of NPC patients. The four DL models used in this study are commercially available systems that were independently developed by different vendors. Inter-model agreement for each structure was quantified using the overall Kappa coefficient and its associated p-value. All p-values were < 0.001 (Supplement 2: Tables S1–S23), indicating that the observed agreement among models was clearly higher than random, although the magnitude of agreement varied across structures. Nonetheless, regions with well-defined anatomical boundaries—such as the body, eyes, and mandible—exhibited almost perfect agreement among the models (Fig. 1). In contrast, lower consistency was observed for complex or low-contrast structures such as the GTV, lenses, pituitary glands, and temporomandibular joints. The remaining ROIs generally showed substantial agreement (0.61 < overall kappa values < 0.80, 0.5 < CI-gen < 0.7).

### Assessment of clinical feasibility

For this section of the analysis, data from the same 30 NPC patients used in the first part were utilized. Through the analysis of two contour metrics, the DSC and RVD, it can be observed that in most OARs, the four DL models have an average DSC delineation value greater than 0.7 and an average RVD value less than 30%, demonstrating excellent contouring performance. However, compared to the manual contours, the four DL models still exhibit marked differences in contours such as the GTV, the lens, optic nerves, pituitary glands, and temporomandibular joints. The worst DSC mean is the GTV, with the mean DSC values less than 0.6 in both DL models. When it comes to pituitary gland, the discrepancy between manual and automated delineations can reach a magnitude of up to four times the reference volume. At the same time, some automatic contouring software may have marked differences in contours such as spinal cord, submandibular glands, temporal lobes and trachea compared to manual contours (Tables 1 and 2).

**Table 1:** Comparison of DSC for GTV and OAR automatic delineation of 30 nasopharyngeal carcinoma patients by four deep learning models.

ROI	DL-1	DL-2	DL-3	DL-4
GTV	**0.588 ± 0.167**		**0.569 ± 0.171**	
Body	0.989 ± 0.012	0.991 ± 0.011	0.994 ± 0.012	0.992 ± 0.012
Brainsteam	0.886 ± 0.034	0.862 ± 0.048	0.866 ± 0.034	0.848 ± 0.034
Esophagus	0.822 ± 0.096	0.782 ± 0.114	0.850 ± 0.096	0.823 ± 0.096
Eye L	0.895 ± 0.055	0.884 ± 0.061	0.860 ± 0.057	0.887 ± 0.063
Eye R	0.896 ± 0.036	0.898 ± 0.027	0.882 ± 0.034	0.881 ± 0.045
Len L	0.721 ± 0.123	**0.535 ± 0.144**	**0.681 ± 0.154**	**0.682 ± 0.114**
Len R	0.735 ± 0.114	**0.611 ± 0.134**	**0.690 ± 0.128**	**0.693 ± 0.099**
Mandible	0.911 ± 0.037	0.878 ± 0.045	0.906 ± 0.037	0.911 ± 0.037
Optic Nerve L	**0.650 ± 0.107**	**0.619 ± 0.123**	**0.690 ± 0.123**	**0.656 ± 0.109**
Optic Nerve R	**0.626 ± 0.106**	**0.695 ± 0.085**	**0.697 ± 0.105**	**0.728 ± 0.085**
Parotid Gland L	0.791 ± 0.118	0.733 ± 0.132	0.813 ± 0.112	0.802 ± 0.118
Parotid Gland R	0.835 ± 0.127	0.794 ± 0.080	0.851 ± 0.118	0.857 ± 0.117
Pituitary Glands	**0.667 ± 0.174**	**0.587 ± 0.215**	**0.603 ± 0.174**	**0.511 ± 0.174**
Spinal Cord	0.726 ± 0.115	0.813 ± 0.075	0.808 ± 0.115	0.815 ± 0.115
Submandibular Glands L	0.752 ± 0.115	0.741 ± 0.128	0.765 ± 0.165	0.742 ± 0.154
Submandibular Glands R	0.784 ± 0.125	0.752 ± 0.148	0.807 ± 0.140	0.777 ± 0.108
Temporal Lobes L	0.857 ± 0.055	**0.671 ± 0.094**	0.799 ± 0.072	0.871 ± 0.075
Temporal Lobes R	0.847 ± 0.060	**0.690 ± 0.088**	0.818 ± 0.077	0.855 ± 0.083
Temporomandibular Joints L	**0.646 ± 0.181**	**0.540 ± 0.129**	**0.579 ± 0.186**	**0.594 ± 0.181**
Temporomandibular Joints R	**0.667 ± 0.143**	**0.605 ± 0.158**	**0.581 ± 0.189**	**0.640 ± 0.173**
Thyroid Gland	0.835 ± 0.089	0.842 ± 0.095	0.799 ± 0.089	0.848 ± 0.089
Trachea	0.856 ± 0.078	0.861 ± 0.071	0.880 ± 0.078	**0.640 ± 0.078**

Average DSC (Dice Similarity Coefficient) with standard deviations for autocontours from DL-1 (AccuContour), DL-2 (RT-Viewer-contour), DL-3 (RT-Mind), and DL-4 (PVmed Contouring). Values with a mean DSC less than 0.7 are shown in bold. ROI (Regions of Interest), GTV (Gross Tumor Volume).

**Table 2:** Comparison of RVD (%) for GTV and OAR automatic delineation of 30 nasopharyngeal carcinoma patients by four deep learning models.

ROI	DL-1	DL-2	DL-3	DL-4
GTV	**41.894 ± 27.278**		**61.530 ± 59.372**	
Body	1.345 ± 1.905	1.042 ± 1.837	0.981 ± 2.222	1.022 ± 2.182
Brainsteam	12.823 ± 10.776	20.077 ± 13.301	11.873 ± 9.868	10.006 ± 5.822
Esophagus	10.088 ± 12.429	11.796 ± 18.033	9.248 ± 13.259	13.675 ± 20.468
Eye L	12.907 ± 13.476	18.044 ± 17.762	25.009 ± 21.168	18.044 ± 17.762
Eye R	12.749 ± 7.934	13.033 ± 7.913	18.042 ± 12.794	18.209 ± 10.150
Len L	**43.570 ± 39.985**	**134.982 ± 101.209**	**57.227 ± 68.725**	**39.058 ± 33.444**
Len R	**47.706 ± 47.876**	**111.263 ± 86.731**	**49.444 ± 56.641**	**27.362 ± 20.868**
Mandible	9.406 ± 6.040	19.249 ± 12.341	14.705 ± 11.666	10.786 ± 8.282
Optic Nerve L	23.592 ± 15.840	**54.437 ± 54.586**	24.924 ± 25.896	25.013 ± 20.970
Optic Nerve R	**31.167 ± 14.494**	**42.996 ± 35.130**	28.051 ± 25.296	20.570 ± 15.868
Parotid Gland L	25.676 ± 25.566	27.219 ± 23.909	25.180 ± 32.223	**38.867 ± 42.865**
Parotid Gland R	15.456 ± 16.576	16.336 ± 18.203	12.684 ± 14.450	17.824 ± 15.925
Pituitary Glands	**59.030 ± 78.293**	**411.000 ± 1688.964**	**125.858 ± 159.979**	**180.919 ± 196.285**
Spinal Cord	**66.928 ± 53.319**	26.422 ± 21.245	**35.345 ± 35.503**	26.771 ± 28.933
Submandibular Glands L	**30.087 ± 31.462**	**31.841 ± 28.700**	**44.013 ± 74.227**	**74.741 ± 79.049**
Submandibular Glands R	20.392 ± 18.029	22.050 ± 16.836	18.684 ± 18.080	**50.053 ± 34.642**
Temporal Lobes L	11.134 ± 9.745	**60.285 ± 39.880**	18.810 ± 13.439	13.763 ± 16.318
Temporal Lobes R	12.144 ± 9.201	**65.883 ± 37.451**	11.708 ± 10.329	14.331 ± 13.907
Temporomandibular Joints L	**38.528 ± 52.675**	**88.366 ± 63.431**	**24.173 ± 15.991**	**50.629 ± 67.870**
Temporomandibular Joints R	**30.867 ± 29.036**	**53.930 ± 44.571**	26.074 ± 20.841	**34.944 ± 25.428**
Thyroid Gland	19.049 ± 21.066	15.534 ± 14.025	**42.925 ± 31.185**	14.536 ± 16.589
Trachea	18.133 ± 15.821	14.160 ± 14.101	13.555 ± 15.744	**96.264 ± 99.486**

Average RVD (Relative Volume Difference) with standard deviations for autocontours from DL-1 (AccuContour), DL-2 (RT-Viewer-contour), DL-3 (RT-Mind), and DL-4 (PVmed Contouring). Values with a mean RVD more than 30% are shown in bold. ROI (Regions of Interest), GTV (Gross Tumor Volume).

The HD95 results showed that the deviations generated by automatic contouring are more severe in the delineation of structures such as the GTV, optic nerves, parotid glands, pituitary glands, temporal lobes, temporomandibular joints and trachea. The GTV contours generated by the two DL models showed marked differences, as the mean HD95 values were greater than 15 mm (Table 3). On the other hand, the ASSD results showed that except for structures such as the GTV, parotid glands, temporal lobes, temporomandibular joints, and trachea, the errors in automatic contours were relatively small, with the mean ASSD value less than 3 mm (Table 4).

**Table 3:** Comparison of HD95 (mm) for GTV and OAR automatic delineation of 30 nasopharyngeal carcinoma patients by four deep learning models.

ROI	DL-1	DL-2	DL-3	DL-4
GTV	**22.330 ± 45.798**		**16.346 ± 9.098**	
Body	2.408 ± 6.205	2.573 ± 7.217	**5.386 ± 21.883**	**5.743 ± 20.225**
Brainsteam	3.778 ± 2.682	4.026 ± 2.612	3.726 ± 1.987	**6.290 ± 3.724**
Esophagus	3.164 ± 2.220	**6.586 ± 11.834**	3.901 ± 6.876	3.075 ± 2.562
Eye L	4.315 ± 10.784	4.753 ± 10.746	4.753 ± 10.746	**5.248 ± 10.670**
Eye R	2.447 ± 0.911	4.066 ± 9.798	3.243 ± 0.997	2.789 ± 0.761
Len L	4.679 ± 11.767	**7.250 ± 15.031**	**5.064 ± 11.434**	**5.270 ± 11.216**
Len R	2.422 ± 1.123	2.872 ± 1.114	3.096 ± 1.531	2.890 ± 1.075
Mandible	2.114 ± 1.081	3.097 ± 1.406	2.762 ± 1.305	2.581 ± 1.999
Optic Nerve L	**6.204 ± 8.476**	**7.036 ± 10.897**	**6.783 ± 11.906**	**5.436 ± 8.302**
Optic Nerve R	**7.074 ± 9.241**	4.508 ± 7.753	2.997 ± 0.659	2.777 ± 0.547
Parotid Gland L	**6.585 ± 3.792**	**22.410 ± 32.224**	**10.443 ± 21.234**	**9.107 ± 5.810**
Parotid Gland R	**5.041 ± 3.852**	**14.209 ± 20.235**	4.784 ± 2.861	**5.720 ± 3.436**
Pituitary Glands	**5.121 ± 12.454**	**5.387 ± 12.889**	**5.292 ± 12.861**	**5.719 ± 12.532**
Spinal Cord	**5.454 ± 10.473**	**6.264 ± 10.413**	4.476 ± 9.891	5.741 ± 10.501
Submandibular Glands L	4.982 ± 2.645	**8.044 ± 13.377**	4.780 ± 3.165	5.714 ± 3.617
Submandibular Glands R	4.643 ± 4.391	4.823 ± 2.627	4.030 ± 3.213	4.500 ± 2.298
Temporal Lobes L	**9.894 ± 13.052**	**28.119 ± 26.703**	**14.877 ± 13.589**	**10.336 ± 13.411**
Temporal Lobes R	**9.075 ± 4.025**	**14.788 ± 5.563**	**12.341 ± 5.481**	**8.906 ± 2.832**
Temporomandibular Joints L	**5.800 ± 3.191**	**13.592 ± 25.069**	**6.426 ± 3.823**	**6.844 ± 4.013**
Temporomandibular Joints R	4.880 ± 2.406	**9.039 ± 18.141**	**5.874 ± 3.322**	**5.418 ± 2.515**
Thyroid Gland	2.989 ± 1.962	3.099 ± 1.650	3.261 ± 1.849	2.603 ± 2.060
Trachea	**11.044 ± 12.430**	**11.964 ± 14.893**	**15.163 ± 18.111**	**29.831 ± 26.663**

Average HD95 (the 95th percentile Hausdorff Distance) with standard deviations for autocontours from DL-1 (AccuContour), DL-2 (RT-Viewer-contour), DL-3 (RT-Mind), and DL-4 (PVmed Contouring). Values with a mean HD95 more than 5 mm are shown in bold. ROI (Regions of Interest), GTV (Gross Tumor Volume).

**Table 4:** Comparison of ASSD (mm) for GTV and OAR automatic delineation of 30 nasopharyngeal carcinoma patients by four deep learning models.

ROI	DL-1	DL-2	DL-3	DL-4
GTV	**6.052 ± 7.533**		**5.628 ± 3.427**	
Body	0.662 ± 1.625	0.855 ± 2.248	1.118 ± 2.899	1.047 ± 2.703
Brainsteam	0.926 ± 0.539	1.158 ± 0.620	1.115 ± 0.685	1.455 ± 0.813
Esophagus	0.767 ± 0.523	1.785 ± 3.102	0.632 ± 0.628	0.753 ± 0.802
Eye L	0.569 ± 0.423	0.750 ± 0.447	0.664 ± 0.411	0.920 ± 0.400
Eye R	0.568 ± 0.277	1.001 ± 2.647	0.752 ± 0.271	0.657 ± 0.250
Len L	0.995 ± 1.831	1.312 ± 0.886	1.321 ± 1.838	1.155 ± 1.771
Len R	0.564 ± 0.357	0.926 ± 0.478	0.759 ± 0.437	0.702 ± 0.299
Mandible	0.455 ± 0.191	0.686 ± 0.286	0.611 ± 0.379	0.526 ± 0.329
Optic Nerve L	1.135 ± 1.257	1.939 ± 4.088	1.656 ± 3.835	1.081 ± 1.246
Optic Nerve R	1.450 ± 0.909	1.341 ± 3.742	0.637 ± 0.251	0.520 ± 0.185
Parotid Gland L	1.948 ± 1.455	**9.117 ± 15.902**	2.896 ± 5.980	2.717 ± 2.076
Parotid Gland R	1.328 ± 1.211	**3.915 ± 9.985**	1.209 ± 0.919	1.312 ± 0.857
Pituitary Glands	2.070 ± 6.718	2.416 ± 6.968	2.385 ± 6.948	2.620 ± 6.755
Spinal Cord	1.644 ± 1.645	1.322 ± 1.475	1.202 ± 1.492	1.385 ± 1.816
Submandibular Glands L	1.755 ± 2.133	2.707 ± 4.744	1.734 ± 2.285	2.176 ± 2.417
Submandibular Glands R	1.250 ± 1.329	1.391 ± 0.941	1.064 ± 1.153	1.264 ± 0.703
Temporal Lobes L	2.466 ± 3.162	**9.568 ± 10.491**	**3.772 ± 3.597**	2.531 ± 3.236
Temporal Lobes R	2.152 ± 0.929	**4.630 ± 2.068**	2.896 ± 1.463	2.082 ± 1.021
Temporomandibular Joints L	1.919 ± 1.439	2.830 ± 1.650	2.429 ± 1.566	2.484 ± 1.590
Temporomandibular Joints R	1.629 ± 0.926	**3.463 ± 7.512**	2.196 ± 1.423	1.828 ± 1.000
Thyroid Gland	0.834 ± 0.643	0.829 ± 0.615	1.060 ± 0.708	0.725 ± 0.674
Trachea	2.217 ± 2.782	2.599 ± 3.435	**3.302 ± 4.199**	**8.460 ± 11.141**

Average ASSD (Average Symmetric Surface Distance) with standard deviations for autocontours from DL-1 (AccuContour), DL-2 (RT-Viewer-contour), DL-3 (RT-Mind), and DL-4 (PVmed Contouring). Values with a mean ASSD more than 3 mm are shown in bold. ROI (Regions of Interest), GTV (Gross Tumor Volume).

### Model architecture

After random division, data from 13 nasopharyngeal carcinoma patients were selected for testing (Table 5). The same ROIs were selected to compute the DSC as the evaluation metric. Results demonstrated that both (Method A) and (Method B) achieved stable and reliable optimization effects for GTV, exhibiting higher means and lower standard deviations.

**Table 5:** Comparison of DSC for GTV and OAR automatic delineation of 12 nasopharyngeal carcinoma patients by four deep learning models and method A and method B.

ROI	DL-1	DL-2	DL-3	DL-4	Method A	Method B
GTV	0.625 ± 0.135		0.581 ± 0.133		**0.664 ± 0.121**	0.659 ± 0.100
Body	0.992 ± 0.001	0.993 ± 0.001	0.999 ± 0.002	0.995 ± 0.002	0.996 ± 0.001	0.991 ± 0.002
Brainsteam	0.886 ± 0.028	0.856 ± 0.033	0.891 ± 0.037	0.810 ± 0.075	0.886 ± 0.024	**0.905 ± 0.019**
Esophagus	0.825 ± 0.018	0.730 ± 0.140	0.920 ± 0.045	0.862 ± 0.050	0.801 ± 0.160	0.885 ± 0.014
Eye L	0.907 ± 0.018	0.876 ± 0.030	0.907 ± 0.041	0.848 ± 0.030	**0.913 ± 0.019**	**0.915 ± 0.012**
Eye R	0.902 ± 0.011	0.883 ± 0.022	0.894 ± 0.026	0.872 ± 0.041	**0.911 ± 0.013**	**0.920 ± 0.014**
Len L	0.693 ± 0.147	0.484 ± 0.122	0.744 ± 0.099	0.721 ± 0.147	0.703 ± 0.093	**0.815 ± 0.110**
Len R	0.689 ± 0.100	0.603 ± 0.098	0.754 ± 0.108	0.729 ± 0.112	0.766 ± 0.053	**0.815 ± 0.087**
Mandible	0.849 ± 0.063	0.803 ± 0.089	0.943 ± 0.038	0.768 ± 0.148	0.902 ± 0.018	0.925 ± 0.023
Optic Nerve L	0.542 ± 0.064	0.533 ± 0.092	0.542 ± 0.126	0.598 ± 0.082	0.559 ± 0.060	0.588 ± 0.169
Optic Nerve R	0.495 ± 0.228	0.701 ± 0.072	0.826 ± 0.080	0.730 ± 0.066	0.610 ± 0.130	0.710 ± 0.073
Parotid Gland L	0.713 ± 0.171	0.690 ± 0.184	0.899 ± 0.079	0.798 ± 0.079	0.783 ± 0.108	0.871 ± 0.048
Parotid Gland R	0.828 ± 0.075	0.809 ± 0.033	0.845 ± 0.293	0.859 ± 0.055	0.811 ± 0.109	**0.892 ± 0.035**
Pituitary Glands	0.623 ± 0.094	0.545 ± 0.130	0.687 ± 0.139	0.720 ± 0.187	0.613 ± 0.071	0.669 ± 0.078
Spinal Cord	0.778 ± 0.098	0.828 ± 0.046	0.892 ± 0.100	0.826 ± 0.070	0.851 ± 0.051	0.872 ± 0.087
Submandibular Glands L	0.756 ± 0.133	0.771 ± 0.174	0.866 ± 0.191	0.810 ± 0.168	0.861 ± 0.128	0.851 ± 0.029
Submandibular Glands R	0.762 ± 0.147	0.728 ± 0.194	0.841 ± 0.184	0.775 ± 0.154	**0.858 ± 0.136**	**0.843 ± 0.061**
Temporal Lobe L	0.893 ± 0.032	0.637 ± 0.157	0.943 ± 0.032	0.816 ± 0.038	0.878 ± 0.032	0.883 ± 0.025
Temporal Lobe R	0.888 ± 0.024	0.688 ± 0.029	0.962 ± 0.037	0.859 ± 0.034	0.888 ± 0.020	0.917 ± 0.025
Temporomandibular Joint L	0.653 ± 0.077	0.576 ± 0.113	0.770 ± 0.087	0.545 ± 0.156	0.758 ± 0.057	**0.791 ± 0.078**
Temporomandibular Joint R	0.653 ± 0.077	0.627 ± 0.098	0.758 ± 0.135	0.512 ± 0.178	**0.765 ± 0.030**	**0.806 ± 0.070**
Thyroid Gland	0.792 ± 0.090	0.748 ± 0.103	0.871 ± 0.133	0.805 ± 0.097	0.753 ± 0.147	0.830 ± 0.047
Trachea	0.819 ± 0.061	0.808 ± 0.072	0.904 ± 0.080	0.711 ± 0.116	0.829 ± 0.055	0.879 ± 0.073

Average DSC (Dice Similarity Coefficient) with standard deviations for autocontours from DL-1 (AccuContour), DL-2 (RT-Viewer-contour), DL-3 (RT-Mind), DL-4 (PVmed Contouring), Method A, and Method B. Values for which Method A or Method B outperform the four deep learning models are shown in bold. ROI (Regions of Interest), GTV (Gross Tumor Volume).

As for the OARs, consistent optimization effects were visibly noted in the brainstem, eyes, lens, and temporomandibular joints. The mean DSC of the brainstem was greater than 0.9. In other OARs, while the outcomes from the proposed models did not demonstrate significant superiority over those derived from the existing DL models, they effectively precluded any significant negative optimization. The least favorable results were commensurate with those achieved by the DL models, thereby rendering them directly applicable in actual segmentation tasks.

Comparatively, the results from Method A and Method B were similar, but with the exception of the GTV and body, the segmentation performance of Method A for the other OARs was slightly inferior to that of Method B. Deep learning proved more effective in learning edge information, offering enhanced improvements particularly for segmentation results of OARs.

## Discussion

The advent of artificial intelligence (AI) in the realm of RT has ushered in a new era of automatic delineation for target volumes and OARs, enhancing both the efficiency and reproducibility of contouring^9^. Despite these advancements, uncertainties persist, particularly in the realm of automatic contouring. Our study provides a comprehensive evaluation of the performance of commercial DL models for OAR contouring in NPC. The use of such models is motivated by the well-known challenges of manual contour delineation, including IOV among clinicians^7,8^. In the present work, however, we did not directly measure manual IOV; instead, our primary focus is on the variability between different DL-based auto-contouring tools and on strategies to mitigate this inter-model variability.

The evaluation of four distinct DL models in our study revealed a relatively poor consistency in the automatic contouring of certain structures, such as the GTV, lens, pituitary glands, and temporomandibular joints. The inconsistency may stem from the inherent low contrast of these structures on CT scans, ill-defined borders, or their small size. In contrast, structures with clear anatomical boundaries, such as the body, eyes, and mandible, showed almost perfect agreement across models. Taken together, these findings suggest that, particularly for small or low-contrast structures, physicians should pay closer attention to the review and, when available, consider integrating multimodal imaging to refine the automatic contours. Moreover, because different commercial DL models are trained on heterogeneous datasets with distinct architectures, appreciable inter-model variability can arise even when they are applied to the same institution and contouring guideline. When multiple auto-contouring tools are used within a clinic, it is therefore important to routinely assess their consistency and to verify that the resulting contours are clinically acceptable before integrating them into routine practice.

Our study also scrutinized the clinical acceptability of automatic contours through established contouring metrics. Small-volume structures tended to exhibit more pronounced variability in DSC and RVD, highlighting the sensitivity of these metrics to even minor geometric perturbations^23^. For instance, the pituitary glands, which may span only 2 or 3 layers of CT images, are particularly susceptible to even minor vertical discrepancies. In most cases, however, the required edits for these structures were relatively limited and did not impose a substantial additional workload on physicians. Methodological work has indicated that HD95 may not be fully adequate for capturing contouring inaccuracies, because discrepancies are often distributed along the contour rather than concentrated in a single outlier segment^22,23^. In our data, elevated HD95 values for certain structures are better interpreted as indicators of boundary uncertainty and as a benchmark for assessing the precision of automatic contouring, rather than as a comprehensive measure of overall contour quality. For the majority of structures, the ASSD between automatic and manual contours typically remained below approximately 3 mm. This is consistent with reports advocating the use of margins on the order of 3 mm when defining planning organs-at-risk volumes (PRVs) in head and neck IMRT, and with the magnitude of observed setup and motion uncertainties in this region^25^. The discrepancies between automatic and manual contours in our cohort are thus likely to be dosimetrically acceptable for many OARs in routine practice. Nevertheless, PRVs are primarily designed to account for setup errors and organ motion and do not explicitly incorporate contouring errors or IOV^26^. Integrating the potential errors from automatic contouring into PRV design emerges as a promising strategy to ensure accurate organ dosimetry, thereby enhancing the reliability and clinical utility of automatic contouring techniques.

Consequently, for critical structures such as the GTV, lens, optic nerves, parotid glands, pituitary glands, temporal lobes, temporomandibular joints, and trachea, it is imperative to rigorously enhance the modification processes. This intensification is vital to address and mitigate the uncertainties inherent in automated contouring techniques. In contrast, for other structures, a straightforward review process proves to be adequate for attaining contours that meet clinical acceptability standards. These considerations can help guide the clinical workflow for applying and reviewing automatic contouring, thereby reducing the effective impact of contouring variability and expediting the overall planning process.

Finally, the two model frameworks proposed in this paper, rooted in image confidence algorithm and deep learning respectively, have demonstrated more stable and favorable performance for several key structures, including the GTV, brainstem, eyes, and lenses, while maintaining performance comparable to the best individual DL models for most other OARs. In Method A, the intersection and union of contours from multiple DL models are used to define a trimap, and an image-based confidence algorithm is applied only within the uncertain region. Regions where the models strongly agree are therefore preserved, whereas isolated discrepancies from any single model are down-weighted unless they are supported by the underlying image features, which reduces the influence of outlier segmentations and makes the final contour less sensitive to occasional model failures. In Method B, the planning CT, a fused contour map that summarizes agreement patterns across the models, and the confidence map from Method A are provided as multi-channel inputs to a nnU-Net–based network. By jointly encoding these complementary channels, the network can learn and correct systematic bias patterns of individual models, while retaining their strengths where they perform well. These mechanisms are consistent with the observed increases in mean DSC and reductions in standard deviation for the GTV and several OARs, as well as with the finding that the fused outputs rarely fall below the performance of the better individual models. Compared with recent approaches^10–13^, our fusion framework achieves DSC that are broadly comparable to, or better than, those reported in similar settings, and does so without requiring additional imaging sequences. Admittedly, rigorous cross-study comparisons remain limited by the absence of universally accepted ground-truth contours and standardized evaluation protocols. Nevertheless, by leveraging both in-house and publicly available datasets for localized training, our framework provides a practical solution to enhance the robustness of automatic contouring.

It should be noted that this study has several limitations. First, all evaluations were performed on a single-institution cohort, and our conclusions have not yet been validated on an external test set. Although the commercial DL models themselves were trained on large multicentre datasets, the robustness and inter-model variability observed here may differ in other settings. Theoretically, the proposed frameworks and findings might be applicable to other clinical centres that follow similar guidelines for target volume delineation, but this requires confirmation in future external and cross-institutional studies. Secondly, although manual contours were used as the reference, they may not represent an absolute ground truth. The constraints in contrast and resolution inherent to medical imaging techniques render the creation of perfect true contours an arduous task at present^27,28^. Therefore, our conclusion should be interpreted as the clinical acceptability of these automatic contours in our institution. The impact of contour variations on dosimetry and treatment outcomes is a more clinically relevant endpoint. However, the relationship between them is not clear^29^. The absence of additional dosimetric assessments should be recognized as a shortcoming of our research.

## Conclusion

The contours generated by DL models still have deficiencies in the application of NPC radiotherapy. In structures such as the GTV, lens, optic nerves, parotid glands, pituitary glands, temporal lobes, temporomandibular joints, and trachea, physicians need to strengthen the review and modification to reduce the impact of contouring variability and delineation uncertainty. Two model frameworks, based on an image confidence algorithm and deep learning respectively, were developed to effectively optimize the contours of the GTV, brainstem, eyes, and lens, while ensuring that performance is not degraded, thereby improving the robustness and reliability of the automatic contouring process.

## Supplementary Information

Below is the link to the electronic supplementary material.

Supplementary Material 1

Supplementary Material 2

## Data Availability

The data and analysis code that support the findings of this study are available from the corresponding authors on reasonable request.
