# Supplementary material for: Consistency evaluation and performance optimization of deep learning-based auto-contouring for nasopharyngeal carcinoma
Source: Sci Rep. 2025 Dec 23;16:3564. doi: 10.1038/s41598-025-33567-6 (PMC12848034; doi:10.1038/s41598-025-33567-6)
Supplement: Supplementary file 1 — Supplementary Material 1 [file 41598_2025_33567_MOESM1_ESM.docx]

| **The generalized CI (CI-gen) is expressed as：**  ${CI\_}_{\mathrm{gen}}=\frac{\sum_{pairsi，j} 〡A_{i}\cap A_{j}〡}{\sum_{pairsi，j} 〡A_{i}\cup A_{j}〡}$ | ^1^ |
| --- | --- |

In the given equation, where Ai and Aj stand for i’th and j’th DL model’s contour. Generally, if it is less than 0.5,the overlap is considered low, when it exceeds 0.7, it is considered acceptable.

| The Dice Similarity Coefficient (DSC) is expressed as:  $\mathrm{DSC}=\frac{2〡A\cap B〡}{〡A〡+〡B〡}$ | ^2^ |
| --- | --- |

The Dice similarity coefficient is defined as the ratio of overlap between two volumes. Its value ranges from 0 (no overlap) to 1 (complete overlap). However, it can only perform a metric comparison between 2 sets of contours. In the given equation, where A stand for reference standards’s contour, and B stand for DL model’s contour. Generally, when DSC is less than 0.5, the overlap is considered low; when it exceeds 0.7, it is considered acceptable.

| The Relative Volume Difference (RVD) is expressed as:  $RVD=\frac{〡A-B〡}{A}\times100\%$ |  |
| --- | --- |

The Relative Volume Difference (RVD) has also been used to evaluate image segmentation performance. It measures the relative volume difference between the volume of the target contour B and the reference contour A.

| The the 95th percentile Hausdorff Distance (HD95) is expressed as:  $95HD(A,B)=perecentile[h(A,B)\cup h(B,A),95th]$  $h(A,B)=\begin{matrix} \max& \min\\ a\in A & b\in B \end{matrix}\vert\vert a-b\vert\vert$  $h(B,A)=\begin{matrix} \max& \min\\ b\in B & a\in A \end{matrix}\vert\vert b-a\vert\vert$ | ^3^ |
| --- | --- |

The Hausdorff distance (HD) is the maximum distance from any point in the target contour to the nearest point in the reference contour. The closer the HD value is to 0, the smaller the difference between the two delineated contours. Therefore, HD is very sensitive to outliers. HD95 is commonly used to evaluate IOV because it is less affected by partial extreme values caused by irregular shapes^3,4^.

| The Average Symmetric Surface Distance (ASSD) is expressed as:  $ASSD=\frac{\sum_{a\in A} \min_{b\in B}d(a,b)+\sum_{b\in B} \min_{a\in A}d(b,a)}{len(A)+len(B)}$ | ^5^ |
| --- | --- |

The ASSD provides the average distance between each point on the contour boundary and the nearest point on the reference contour boundary, representing the average distance the contour boundary must move^6^.

**Method A**

The proposed algorithm combines multiple DL models with distinct architectures and training datasets with a medical image confidence algorithm for ROI boundary refinement. First, each DL model individually predicts a binary segmentation mask for the ROI. These predictions are then combined by computing the voxel-wise intersection and union of the masks. Because the individual models are developed independently by different vendors and trained on different datasets, their outputs are expected to be complementary rather than identical. Aggregating their outputs via intersection and union allows the algorithm to highlight regions where the models consistently agree and to localize areas of disagreement that require further refinement. Voxels inside the intersection of the segmentation masks are considered to definitively belong to the ROI, whereas voxels outside the union are considered to definitively not belong to the ROI; all remaining voxels constitute an uncertain region.

A trimap is then generated from the intersection and union masks, assigning voxels to foreground, background, or unknown classes. This trimap serves as the initial segmentation for the medical image confidence algorithm, which is used to identify voxels belonging to the foreground, background, and unknown regions of the ROI. The confidence algorithm^7^ computes a three-dimensional spatial correlation matrix for voxels in the feature space of the planning CT and, for each voxel in the uncertain region, estimates a confidence value between 0 and 1 that it belongs to the ROI based on its similarity to foreground and background voxels in the trimap.

In practice, the union of the ROI segmentation results from all DL models is used as the maximum effective region for computation. All inputs, including the ROI masks, trimap, confidence map, and CT images, are cropped and aligned to this union region. In the final stage, each voxel value in the confidence map is a floating-point number between 0 and 1. A threshold is applied to the confidence map to separate foreground from background in the final segmentation. To determine an appropriate threshold for each ROI, the threshold is linearly varied over the range [0, 1] on the training set, and the Dice similarity coefficient (DSC) between the resulting segmentation and the corresponding manual contour is calculated. The optimal threshold value for each ROI is chosen as the one that yields the highest DSC on the training data and is then fixed for all test cases. This procedure defines the most appropriate division threshold for the confidence map of that ROI.

**Method B**

The second proposed framework is based on the nnU-Net deep learning architecture, with the main innovation lying in the design of the model input. In addition to the planning CT, a feature map derived from the fusion of contours generated by multiple DL models is used. To construct this map, the contours from all DL models are first reoriented, resampled, and aligned within a unified coordinate system. The binary masks are then aggregated voxel-wise; regions where more models include the voxel in the ROI have higher values and thus higher weight for that ROI. This aggregated mask serves as a fused contour map that encodes the agreement pattern among models. In addition, a confidence map generated from the planning CT and DL model contours using Method A is included. The planning CT images, fused contour map, and confidence map are concatenated to form a multi-channel input to the fusion network.

For training, the target in-plane voxel spacing for CT images is set to 1.31 mm × 1.31 mm, and image intensities are clipped to the range −1000 to 1000 HU. Data augmentation techniques are applied to the training samples, including scaling (range 0.7–1.3, probability 0.4), rotation (range −30° to 30°, probability 0.4), additive Gaussian noise (σ range 0–0.1, probability 0.2), Gaussian blur (σ range 0.25–1.5, probability 0.2), and nonlinear intensity transformations (5 mapping segments, probability 0.2). The loss function is a combination of Dice loss and cross-entropy loss. The Adam optimizer is used with an initial learning rate of 0.01 and a weight decay of 3 × 10⁻⁵. Training is conducted for 300 epochs, each consisting of 250 training iterations and 50 validation iterations. For the deep learning component, the dataset is randomly divided into a training set of 48 cases and an independent test set of 13 cases.

By providing the planning CT, fused contour map, and confidence map as multi-channel input, Method B enables the nnU-Net–based network to learn from both anatomical information and the agreement pattern among the DL models, as well as from the confidence estimation produced by Method A. This design allows the network to correct systematic biases in individual model outputs while preserving their strengths.

**Reference**

1. Kouwenhoven, E., Giezen, M. & Struikmans, H. Measuring the similarity of target volume delineations independent of the number of observers. *Phys. Med. Biol.* **54**, 2863–2873 (2009).

2. Dice, L. R. Measures of the Amount of Ecologic Association Between Species. *Ecology* **26**, 297–302 (1945).

3. Huttenlocher, D. P., Klanderman, G. A. & Rucklidge, W. J. Comparing images using the hausdorff distance. *IEEE Trans. Pattern Anal. Mach. Intell.* **15**, 850–863 (1993).

4. Guzene, L. *et al.* Assessing Interobserver Variability in the Delineation of Structures in Radiation Oncology: A Systematic Review. *Int. J. Radiat. Oncol. Biol. Phys.* **115**, 1047–1060 (2023).

5. Taha, A. A. & Hanbury, A. Metrics for evaluating 3D medical image segmentation: analysis, selection, and tool. *BMC Med. Imaging* **15**, 29 (2015).

6. Cannon, J., Bownes, P., Mason, J. & Cooper, R. UK audit of target volume and organ at risk delineation and dose optimisation for cervix radiotherapy treatments. *Br. J. Radiol.* **93**, 20190897 (2020).

7. Yang, L. *et al.* Multimodal Image Confidence: A Novel Method for Tumor and Organ Boundary Representation. *Int. J. Radiat. Oncol. Biol. Phys.* **121**, 558–569 (2025).
