# Supplementary material for: Consistency evaluation and performance optimization of deep learning-based auto-contouring for nasopharyngeal carcinoma
Source: Sci Rep. 2025 Dec 23;16:3564. doi: 10.1038/s41598-025-33567-6 (PMC12848034; doi:10.1038/s41598-025-33567-6)
Supplement: Supplementary file 2 — Supplementary Material 2 [file 41598_2025_33567_MOESM2_ESM.docx]

| Case | volume | Intersection volume | Union volume | Sensitivity | Specificity | p-value: | Overall kappa: | Mean±SD | CI-gen | Mean±SD |
| --- | --- | --- | --- | --- | --- | --- | --- | --- | --- | --- |
| 1 | 37136.509±911.407 | 35648.811 | 38516.033 | 0.979±0.020 | 0.998±0.003 | ＜0.001 | 0.968 | 0.988±0.005 | 0.957 | 0.982±0.009 |
| 2 | 10510.471±63.035 | 10364.499 | 10638.738 | 0.993±0.005 | 0.999±0.001 | ＜0.001 | 0.990 |  | 0.986 |  |
| 3 | 11632.362±100.208 | 11435.302 | 11867.670 | 0.993±0.004 | 0.999±0.001 | ＜0.001 | 0.988 |  | 0.980 |  |
| 4 | 11711.604±72.921 | 11555.785 | 11861.434 | 0.994±0.004 | 0.999±0.001 | ＜0.001 | 0.990 |  | 0.986 |  |
| 5 | 19413.533±116.293 | 19191.407 | 19619.906 | 0.995±0.004 | 0.999±0.001 | ＜0.001 | 0.991 |  | 0.988 |  |
| 6 | 14367.751±82.553 | 14188.842 | 14531.312 | 0.994±0.004 | 0.999±0.001 | ＜0.001 | 0.991 |  | 0.987 |  |
| 7 | 14248.489±99.925 | 14047.051 | 14436.847 | 0.993±0.005 | 0.999±0.001 | ＜0.001 | 0.990 |  | 0.985 |  |
| 8 | 9817.564±73.418 | 9650.511 | 9997.739 | 0.992±0.004 | 0.999±0.001 | ＜0.001 | 0.988 |  | 0.981 |  |
| 9 | 12079.980±62.982 | 11926.209 | 12233.149 | 0.994±0.003 | 0.999±0.001 | ＜0.001 | 0.991 |  | 0.986 |  |
| 10 | 12157.453±81.641 | 11986.403 | 12306.668 | 0.993±0.005 | 0.999±0.001 | ＜0.001 | 0.990 |  | 0.986 |  |
| 11 | 13082.526±82.088 | 12891.711 | 13264.849 | 0.993±0.004 | 0.999±0.001 | ＜0.001 | 0.989 |  | 0.984 |  |
| 12 | 11293.826±73.481 | 11125.875 | 11463.349 | 0.993±0.004 | 0.999±0.001 | ＜0.001 | 0.989 |  | 0.984 |  |
| 13 | 13063.215±80.016 | 12880.563 | 13241.824 | 0.994±0.004 | 0.999±0.001 | ＜0.001 | 0.990 |  | 0.985 |  |
| 14 | 11084.987±251.660 | 10647.505 | 11336.903 | 0.985±0.021 | 0.999±0.001 | ＜0.001 | 0.978 |  | 0.968 |  |
| 15 | 13331.741±87.107 | 13149.564 | 13515.840 | 0.994±0.004 | 0.999±0.001 | ＜0.001 | 0.990 |  | 0.985 |  |
| 16 | 14310.116±108.027 | 14095.830 | 14507.809 | 0.993±0.006 | 0.999±0.001 | ＜0.001 | 0.989 |  | 0.984 |  |
| 17 | 8524.882±79.298 | 8371.520 | 8709.266 | 0.993±0.005 | 0.999±0.001 | ＜0.001 | 0.987 |  | 0.979 |  |
| 18 | 14677.170±82.968 | 14493.961 | 14854.877 | 0.994±0.004 | 0.999±0.001 | ＜0.001 | 0.990 |  | 0.987 |  |
| 19 | 11642.760±85.253 | 11462.874 | 11816.077 | 0.993±0.005 | 0.999±0.001 | ＜0.001 | 0.989 |  | 0.983 |  |
| 20 | 10550.059±66.572 | 10399.629 | 10682.887 | 0.993±0.005 | 0.999±0.000 | ＜0.001 | 0.990 |  | 0.985 |  |
| 21 | 15572.348±102.551 | 15370.785 | 15771.781 | 0.994±0.004 | 0.999±0.001 | ＜0.001 | 0.990 |  | 0.986 |  |
| 22 | 13052.443±84.119 | 12863.887 | 13232.341 | 0.993±0.005 | 0.999±0.001 | ＜0.001 | 0.989 |  | 0.985 |  |
| 23 | 13040.835±90.914 | 12862.579 | 13208.338 | 0.994±0.005 | 0.999±0.001 | ＜0.001 | 0.990 |  | 0.986 |  |
| 24 | 7681.158±47.912 | 7556.042 | 7806.246 | 0.993±0.004 | 0.999±0.001 | ＜0.001 | 0.988 |  | 0.982 |  |
| 25 | 13674.708±88.270 | 13499.660 | 13835.409 | 0.994±0.005 | 0.999±0.001 | ＜0.001 | 0.991 |  | 0.987 |  |
| 26 | 9622.175±56.788 | 9470.356 | 9770.064 | 0.993±0.004 | 0.999±0.001 | ＜0.001 | 0.989 |  | 0.983 |  |
| 27 | 10366.981±67.484 | 10200.087 | 10527.018 | 0.993±0.004 | 0.999±0.001 | ＜0.001 | 0.989 |  | 0.983 |  |
| 28 | 11970.468±87.866 | 11825.822 | 12100.571 | 0.996±0.005 | 0.999±0.001 | ＜0.001 | 0.990 |  | 0.946 |  |
| 29 | 10848.545±59.990 | 10706.765 | 10973.632 | 0.994±0.005 | 0.999±0.000 | ＜0.001 | 0.991 |  | 0.986 |  |
| 30 | 10695.110±62.199 | 10552.762 | 10825.124 | 0.994±0.004 | 0.999±0.001 | ＜0.001 | 0.990 |  | 0.986 |  |

TableS1：Body

The mean volumes (± SD) of the auto-contours of the Body for each patient, as well as the intersection and union volumes, sensitivity, specificity, the overall Kappa coefficient with its p-value (H₀: κ = 0), and the generalized conformity index (CI-gen) for each case. The overall Kappa coefficient with its p-value (H₀: κ = 0); p < 0.001 indicates agreement significantly higher than random.

| Case | volume | Intersection volume | Union volume | Sensitivity | Specificity | p-value: | Overall kappa: | Mean±SD | CI-gen | Mean±SD |
| --- | --- | --- | --- | --- | --- | --- | --- | --- | --- | --- |
| 1 | 24.179±4.595 | 17.034 | 33.744 | 0.907±0.082 | 0.955±0.055 | ＜0.001 | 0.743 | 0.761±0.030 | 0.692 | 0.710±0.035 |
| 2 | 21.471±2.646 | 14.183 | 30.735 | 0.885±0.028 | 0.954±0.049 | ＜0.001 | 0.717 |  | 0.656 |  |
| 3 | 24.931±1.553 | 18.539 | 32.665 | 0.906±0.018 | 0.973±0.021 | ＜0.001 | 0.796 |  | 0.736 |  |
| 4 | 27.960±2.464 | 18.036 | 39.225 | 0.880±0.063 | 0.960±0.045 | ＜0.001 | 0.723 |  | 0.666 |  |
| 5 | 23.804±3.574 | 17.666 | 31.326 | 0.917±0.059 | 0.960±0.045 | ＜0.001 | 0.778 |  | 0.736 |  |
| 6 | 29.043±2.471 | 22.974 | 37.511 | 0.940±0.014 | 0.955±0.039 | ＜0.001 | 0.800 |  | 0.765 |  |
| 7 | 25.799±1.676 | 19.066 | 34.301 | 0.920±0.037 | 0.951±0.041 | ＜0.001 | 0.767 |  | 0.725 |  |
| 8 | 24.134±1.962 | 17.805 | 31.914 | 0.883±0.036 | 0.975±0.026 | ＜0.001 | 0.784 |  | 0.720 |  |
| 9 | 26.356±3.152 | 19.461 | 36.180 | 0.896±0.058 | 0.967±0.033 | ＜0.001 | 0.770 |  | 0.704 |  |
| 10 | 24.424±2.852 | 18.140 | 34.074 | 0.908±0.033 | 0.956±0.041 | ＜0.001 | 0.755 |  | 0.699 |  |
| 11 | 27.705±3.972 | 19.955 | 36.407 | 0.896±0.065 | 0.965±0.043 | ＜0.001 | 0.768 |  | 0.724 |  |
| 12 | 23.081±1.150 | 14.608 | 30.844 | 0.861±0.107 | 0.964±0.041 | ＜0.001 | 0.720 |  | 0.678 |  |
| 13 | 23.315±1.554 | 14.202 | 33.287 | 0.870±0.105 | 0.948±0.050 | ＜0.001 | 0.686 |  | 0.640 |  |
| 14 | 24.239±3.326 | 18.103 | 32.349 | 0.901±0.052 | 0.973±0.028 | ＜0.001 | 0.791 |  | 0.724 |  |
| 15 | 26.750±4.202 | 20.527 | 36.043 | 0.938±0.043 | 0.953±0.054 | ＜0.001 | 0.780 |  | 0.734 |  |
| 16 | 28.672±3.784 | 20.998 | 38.937 | 0.928±0.058 | 0.945±0.052 | ＜0.001 | 0.753 |  | 0.718 |  |
| 17 | 22.120±2.564 | 15.016 | 30.786 | 0.902±0.022 | 0.959±0.044 | ＜0.001 | 0.744 |  | 0.683 |  |
| 18 | 26.731±3.507 | 21.096 | 35.349 | 0.938±0.045 | 0.958±0.046 | ＜0.001 | 0.798 |  | 0.751 |  |
| 19 | 23.788±2.574 | 17.569 | 33.026 | 0.916±0.044 | 0.947±0.046 | ＜0.001 | 0.746 |  | 0.703 |  |
| 20 | 22.835±3.103 | 16.625 | 31.119 | 0.921±0.061 | 0.953±0.033 | ＜0.001 | 0.761 |  | 0.710 |  |
| 21 | 25.051±3.499 | 18.717 | 32.650 | 0.917±0.055 | 0.959±0.042 | ＜0.001 | 0.780 |  | 0.741 |  |
| 22 | 26.173±1.948 | 17.672 | 38.077 | 0.900±0.062 | 0.952±0.035 | ＜0.001 | 0.718 |  | 0.659 |  |
| 23 | 26.613±3.310 | 18.973 | 36.828 | 0.885±0.033 | 0.955±0.048 | ＜0.001 | 0.736 |  | 0.688 |  |
| 24 | 22.291±2.185 | 16.961 | 28.752 | 0.896±0.067 | 0.967±0.040 | ＜0.001 | 0.785 |  | 0.744 |  |
| 25 | 25.779±2.123 | 19.062 | 34.031 | 0.915±0.037 | 0.952±0.053 | ＜0.001 | 0.764 |  | 0.731 |  |
| 26 | 27.187±4.327 | 19.517 | 38.203 | 0.923±0.052 | 0.955±0.052 | ＜0.001 | 0.753 |  | 0.693 |  |
| 27 | 24.796±1.603 | 18.717 | 32.178 | 0.931±0.021 | 0.950±0.044 | ＜0.001 | 0.778 |  | 0.739 |  |
| 28 | 24.956±1.262 | 19.618 | 30.641 | 0.925±0.030 | 0.959±0.031 | ＜0.001 | 0.778 |  | 0.648 |  |
| 29 | 28.978±3.039 | 23.380 | 36.933 | 0.938±0.041 | 0.963±0.036 | ＜0.001 | 0.818 |  | 0.777 |  |
| 30 | 29.810±4.253 | 21.457 | 40.499 | 0.905±0.082 | 0.955±0.046 | ＜0.001 | 0.753 |  | 0.705 |  |

TableS2：Brainsteam

The mean volumes (± SD) of the auto-contours of the Brainsteam for each patient, as well as the intersection and union volumes, sensitivity, specificity, the overall Kappa coefficient with its p-value (H₀: κ = 0), and the generalized conformity index (CI-gen) for each case. The overall Kappa coefficient with its p-value (H₀: κ = 0); p < 0.001 indicates agreement significantly higher than random.

| Case | volume | Intersection volume | Union volume | Sensitivity | Specificity | p-value: | Overall kappa: | Mean±SD | CI-gen | Mean±SD |
| --- | --- | --- | --- | --- | --- | --- | --- | --- | --- | --- |
| 1 | 31.824±1.871 | 17.519 | 45.921 | 0.819±0.078 | 0.996±0.002 | ＜0.001 | 0.746 | 0.778±0.043 | 0.612 | 0.651±0.069 |
| 2 | 13.365±1.169 | 8.163 | 18.456 | 0.834±0.059 | 0.995±0.002 | ＜0.001 | 0.775 |  | 0.653 |  |
| 3 | 14.289±1.973 | 5.716 | 22.608 | 0.753±0.099 | 0.991±0.004 | ＜0.001 | 0.652 |  | 0.514 |  |
| 4 | 13.930±0.762 | 10.362 | 18.052 | 0.889±0.043 | 0.990±0.005 | ＜0.001 | 0.825 |  | 0.737 |  |
| 5 | 16.918±0.569 | 10.402 | 23.622 | 0.825±0.031 | 0.993±0.003 | ＜0.001 | 0.763 |  | 0.603 |  |
| 6 | 15.875±0.796 | 9.954 | 21.822 | 0.844±0.049 | 0.994±0.003 | ＜0.001 | 0.781 |  | 0.663 |  |
| 7 | 15.000±0.872 | 10.367 | 19.802 | 0.873±0.036 | 0.985±0.005 | ＜0.001 | 0.790 |  | 0.708 |  |
| 8 | 17.592±2.354 | 11.409 | 24.284 | 0.855±0.070 | 0.993±0.006 | ＜0.001 | 0.783 |  | 0.669 |  |
| 9 | 18.669±0.636 | 13.967 | 23.219 | 0.891±0.042 | 0.993±0.003 | ＜0.001 | 0.843 |  | 0.761 |  |
| 10 | 17.043±1.541 | 11.439 | 23.558 | 0.858±0.032 | 0.995±0.005 | ＜0.001 | 0.794 |  | 0.675 |  |
| 11 | 19.009±1.919 | 11.996 | 25.978 | 0.845±0.070 | 0.994±0.003 | ＜0.001 | 0.783 |  | 0.667 |  |
| 12 | 13.503±0.677 | 8.677 | 18.710 | 0.845±0.036 | 0.995±0.001 | ＜0.001 | 0.784 |  | 0.663 |  |
| 13 | 36.969±0.608 | 25.871 | 47.225 | 0.873±0.032 | 0.996±0.003 | ＜0.001 | 0.830 |  | 0.729 |  |
| 14 | 12.073±0.942 | 6.837 | 17.833 | 0.810±0.026 | 0.994±0.004 | ＜0.001 | 0.736 |  | 0.601 |  |
| 15 | 18.263±1.535 | 11.484 | 25.845 | 0.828±0.053 | 0.993±0.003 | ＜0.001 | 0.763 |  | 0.642 |  |
| 16 | 15.112±1.724 | 10.494 | 20.596 | 0.865±0.064 | 0.995±0.004 | ＜0.001 | 0.804 |  | 0.690 |  |
| 17 | 11.963±1.036 | 7.330 | 16.801 | 0.842±0.050 | 0.992±0.008 | ＜0.001 | 0.764 |  | 0.647 |  |
| 18 | 19.696±1.715 | 12.966 | 27.814 | 0.850±0.028 | 0.994±0.005 | ＜0.001 | 0.779 |  | 0.658 |  |
| 19 | 15.596±3.455 | 7.906 | 22.120 | 0.802±0.179 | 0.996±0.001 | ＜0.001 | 0.739 |  | 0.603 |  |
| 20 | 14.211±0.376 | 9.091 | 19.285 | 0.844±0.034 | 0.996±0.002 | ＜0.001 | 0.793 |  | 0.672 |  |
| 21 | 16.468±1.042 | 10.341 | 23.170 | 0.839±0.052 | 0.994±0.003 | ＜0.001 | 0.771 |  | 0.649 |  |
| 22 | 16.642±0.320 | 9.966 | 22.979 | 0.830±0.045 | 0.993±0.004 | ＜0.001 | 0.765 |  | 0.649 |  |
| 23 | 22.858±0.975 | 15.243 | 30.444 | 0.866±0.038 | 0.993±0.006 | ＜0.001 | 0.800 |  | 0.696 |  |
| 24 | 10.400±0.282 | 7.225 | 13.661 | 0.879±0.056 | 0.991±0.006 | ＜0.001 | 0.811 |  | 0.714 |  |
| 25 | 19.715±0.674 | 12.881 | 26.663 | 0.847±0.033 | 0.996±0.002 | ＜0.001 | 0.796 |  | 0.678 |  |
| 26 | 12.893±0.685 | 8.702 | 16.803 | 0.862±0.035 | 0.995±0.003 | ＜0.001 | 0.813 |  | 0.708 |  |
| 27 | 10.114±1.654 | 4.593 | 15.825 | 0.771±0.116 | 0.995±0.003 | ＜0.001 | 0.683 |  | 0.536 |  |
| 28 | 15.156±0.230 | 12.191 | 18.150 | 0.927±0.013 | 0.993±0.002 | ＜0.001 | 0.854 |  | 0.436 |  |
| 29 | 14.334±0.400 | 9.860 | 18.931 | 0.871±0.026 | 0.995±0.003 | ＜0.001 | 0.814 |  | 0.708 |  |
| 30 | 16.402±4.210 | 10.049 | 26.246 | 0.852±0.035 | 0.988±0.019 | ＜0.001 | 0.716 |  | 0.583 |  |

TableS3：Esophagus

The mean volumes (± SD) of the auto-contours of the Esophagus for each patient, as well as the intersection and union volumes, sensitivity, specificity, the overall Kappa coefficient with its p-value (H₀: κ = 0), and the generalized conformity index (CI-gen) for each case. The overall Kappa coefficient with its p-value (H₀: κ = 0); p < 0.001 indicates agreement significantly higher than random.

| Case | volume | Intersection volume | Union volume | Sensitivity | Specificity | p-value: | Overall kappa: | Mean±SD | CI-gen | Mean±SD |
| --- | --- | --- | --- | --- | --- | --- | --- | --- | --- | --- |
| 1 | 8.100±1.215 | 6.595 | 9.879 | 0.932±0.067 | 0.963±0.067 | ＜0.001 | 0.817 | 0.785±0.076 | 0.804 | 0.785±0.029 |
| 2 | 8.469±1.493 | 6.620 | 10.786 | 0.931±0.062 | 0.940±0.062 | ＜0.001 | 0.769 |  | 0.768 |  |
| 3 | 10.025±0.867 | 7.891 | 12.054 | 0.913±0.066 | 0.961±0.066 | ＜0.001 | 0.791 |  | 0.794 |  |
| 4 | 7.932±1.280 | 6.222 | 9.807 | 0.921±0.077 | 0.953±0.077 | ＜0.001 | 0.784 |  | 0.783 |  |
| 5 | 8.660±1.293 | 7.018 | 10.611 | 0.921±0.067 | 0.969±0.067 | ＜0.001 | 0.819 |  | 0.797 |  |
| 6 | 10.368±1.364 | 8.279 | 12.708 | 0.928±0.063 | 0.954±0.063 | ＜0.001 | 0.802 |  | 0.790 |  |
| 7 | 9.683±1.183 | 7.952 | 11.492 | 0.923±0.065 | 0.970±0.065 | ＜0.001 | 0.826 |  | 0.817 |  |
| 8 | 8.276±1.069 | 6.398 | 10.086 | 0.910±0.062 | 0.967±0.062 | ＜0.001 | 0.794 |  | 0.785 |  |
| 9 | 6.088±3.986 | 0.295 | 9.771 | 0.714±0.447 | 0.954±0.447 | ＜0.001 | 0.395 |  | 0.785 |  |
| 10 | 9.604±1.665 | 7.096 | 12.115 | 0.895±0.099 | 0.962±0.099 | ＜0.001 | 0.764 |  | 0.751 |  |
| 11 | 8.664±0.936 | 7.107 | 10.262 | 0.931±0.056 | 0.962±0.056 | ＜0.001 | 0.818 |  | 0.820 |  |
| 12 | 10.275±1.327 | 7.261 | 14.698 | 0.871±0.121 | 0.986±0.121 | ＜0.001 | 0.774 |  | 0.669 |  |
| 13 | 9.536±1.021 | 7.668 | 11.294 | 0.924±0.062 | 0.964±0.062 | ＜0.001 | 0.813 |  | 0.811 |  |
| 14 | 9.306±0.950 | 7.556 | 11.080 | 0.935±0.057 | 0.958±0.057 | ＜0.001 | 0.819 |  | 0.813 |  |
| 15 | 8.648±1.348 | 6.525 | 10.562 | 0.890±0.098 | 0.971±0.098 | ＜0.001 | 0.773 |  | 0.771 |  |
| 16 | 9.032±1.057 | 7.484 | 10.710 | 0.930±0.054 | 0.962±0.054 | ＜0.001 | 0.820 |  | 0.821 |  |
| 17 | 9.949±1.146 | 7.874 | 11.875 | 0.899±0.069 | 0.975±0.069 | ＜0.001 | 0.802 |  | 0.796 |  |
| 18 | 8.044±1.217 | 6.290 | 10.095 | 0.925±0.068 | 0.952±0.068 | ＜0.001 | 0.786 |  | 0.774 |  |
| 19 | 8.578±1.510 | 6.553 | 10.672 | 0.909±0.098 | 0.965±0.098 | ＜0.001 | 0.787 |  | 0.772 |  |
| 20 | 9.359±1.238 | 7.387 | 11.436 | 0.921±0.067 | 0.965±0.067 | ＜0.001 | 0.810 |  | 0.790 |  |
| 21 | 8.806±1.370 | 6.648 | 11.048 | 0.898±0.080 | 0.957±0.080 | ＜0.001 | 0.765 |  | 0.757 |  |
| 22 | 7.513±1.211 | 5.635 | 9.260 | 0.902±0.088 | 0.969±0.088 | ＜0.001 | 0.785 |  | 0.770 |  |
| 23 | 8.424±1.252 | 6.628 | 10.416 | 0.926±0.060 | 0.965±0.060 | ＜0.001 | 0.810 |  | 0.787 |  |
| 24 | 9.219±0.935 | 7.520 | 10.829 | 0.926±0.060 | 0.968±0.060 | ＜0.001 | 0.825 |  | 0.820 |  |
| 25 | 9.212±1.288 | 7.358 | 11.348 | 0.936±0.056 | 0.957±0.056 | ＜0.001 | 0.808 |  | 0.794 |  |
| 26 | 8.452±0.990 | 6.593 | 10.144 | 0.911±0.065 | 0.969±0.065 | ＜0.001 | 0.800 |  | 0.795 |  |
| 27 | 7.875±1.187 | 6.039 | 9.737 | 0.898±0.085 | 0.962±0.085 | ＜0.001 | 0.779 |  | 0.768 |  |
| 28 | 9.109±1.346 | 7.112 | 11.477 | 0.917±0.069 | 0.956±0.069 | ＜0.001 | 0.787 |  | 0.769 |  |
| 29 | 8.881±1.157 | 7.090 | 10.787 | 0.911±0.067 | 0.970±0.067 | ＜0.001 | 0.810 |  | 0.794 |  |
| 30 | 9.050±1.220 | 7.112 | 10.963 | 0.917±0.068 | 0.969±0.068 | ＜0.001 | 0.810 |  | 0.795 |  |

TableS4：Eye L

The mean volumes (± SD) of the auto-contours of the Eye-L for each patient, as well as the intersection and union volumes, sensitivity, specificity, the overall Kappa coefficient with its p-value (H₀: κ = 0), and the generalized conformity index (CI-gen) for each case. The overall Kappa coefficient with its p-value (H₀: κ = 0); p < 0.001 indicates agreement significantly higher than random.

| Case | volume | Intersection volume | Union volume | Sensitivity | Specificity | p-value: | Overall kappa: | Mean±SD | CI-gen | Mean±SD |
| --- | --- | --- | --- | --- | --- | --- | --- | --- | --- | --- |
| 1 | 8.226±1.193 | 6.762 | 10.140 | 0.937±0.053 | 0.950±0.053 | ＜0.001 | 0.804 | 0.792±0.023 | 0.800 | 0.780±0.021 |
| 2 | 8.402±1.642 | 6.538 | 10.946 | 0.937±0.054 | 0.944±0.054 | ＜0.001 | 0.778 |  | 0.755 |  |
| 3 | 9.883±1.129 | 7.543 | 12.049 | 0.902±0.062 | 0.960±0.062 | ＜0.001 | 0.774 |  | 0.777 |  |
| 4 | 8.066±1.130 | 6.523 | 9.721 | 0.918±0.076 | 0.967±0.076 | ＜0.001 | 0.810 |  | 0.804 |  |
| 5 | 8.904±1.137 | 7.254 | 10.836 | 0.932±0.046 | 0.965±0.046 | ＜0.001 | 0.823 |  | 0.805 |  |
| 6 | 10.714±1.471 | 8.713 | 13.127 | 0.941±0.043 | 0.952±0.043 | ＜0.001 | 0.809 |  | 0.801 |  |
| 7 | 9.614±1.416 | 7.853 | 11.763 | 0.924±0.062 | 0.967±0.062 | ＜0.001 | 0.820 |  | 0.800 |  |
| 8 | 7.849±1.235 | 6.138 | 9.816 | 0.917±0.059 | 0.964±0.059 | ＜0.001 | 0.799 |  | 0.776 |  |
| 9 | 7.753±1.459 | 5.671 | 9.880 | 0.887±0.099 | 0.960±0.099 | ＜0.001 | 0.747 |  | 0.742 |  |
| 10 | 9.289±1.591 | 7.245 | 11.737 | 0.916±0.061 | 0.955±0.061 | ＜0.001 | 0.782 |  | 0.768 |  |
| 11 | 8.408±1.225 | 6.780 | 10.468 | 0.937±0.043 | 0.959±0.043 | ＜0.001 | 0.815 |  | 0.789 |  |
| 12 | 9.030±1.662 | 6.930 | 11.628 | 0.886±0.086 | 0.965±0.086 | ＜0.001 | 0.770 |  | 0.744 |  |
| 13 | 9.742±1.238 | 7.437 | 11.862 | 0.902±0.070 | 0.971±0.070 | ＜0.001 | 0.795 |  | 0.779 |  |
| 14 | 9.332±0.919 | 7.638 | 11.111 | 0.929±0.050 | 0.968±0.050 | ＜0.001 | 0.832 |  | 0.814 |  |
| 15 | 9.201±1.261 | 6.948 | 11.420 | 0.922±0.075 | 0.956±0.075 | ＜0.001 | 0.786 |  | 0.771 |  |
| 16 | 8.732±1.399 | 6.715 | 10.946 | 0.908±0.069 | 0.956±0.069 | ＜0.001 | 0.774 |  | 0.767 |  |
| 17 | 9.650±1.322 | 7.504 | 11.996 | 0.904±0.064 | 0.958±0.064 | ＜0.001 | 0.779 |  | 0.770 |  |
| 18 | 8.049±1.305 | 6.090 | 9.945 | 0.915±0.104 | 0.956±0.104 | ＜0.001 | 0.777 |  | 0.772 |  |
| 19 | 8.677±1.445 | 6.811 | 10.915 | 0.914±0.062 | 0.955±0.062 | ＜0.001 | 0.778 |  | 0.773 |  |
| 20 | 9.385±1.340 | 7.477 | 11.536 | 0.915±0.064 | 0.960±0.064 | ＜0.001 | 0.798 |  | 0.785 |  |
| 21 | 8.742±1.331 | 6.971 | 10.859 | 0.916±0.060 | 0.964±0.060 | ＜0.001 | 0.802 |  | 0.783 |  |
| 22 | 7.656±1.212 | 5.408 | 9.566 | 0.884±0.094 | 0.968±0.094 | ＜0.001 | 0.754 |  | 0.744 |  |
| 23 | 8.672±1.006 | 6.985 | 10.284 | 0.916±0.070 | 0.969±0.070 | ＜0.001 | 0.811 |  | 0.810 |  |
| 24 | 8.905±1.122 | 6.889 | 10.804 | 0.918±0.067 | 0.960±0.067 | ＜0.001 | 0.790 |  | 0.789 |  |
| 25 | 9.224±1.403 | 7.468 | 11.307 | 0.909±0.078 | 0.965±0.078 | ＜0.001 | 0.799 |  | 0.791 |  |
| 26 | 8.191±1.323 | 6.008 | 10.268 | 0.888±0.083 | 0.960±0.083 | ＜0.001 | 0.746 |  | 0.751 |  |
| 27 | 8.088±1.191 | 6.223 | 10.305 | 0.933±0.045 | 0.936±0.045 | ＜0.001 | 0.767 |  | 0.760 |  |
| 28 | 9.317±1.306 | 7.320 | 11.579 | 0.918±0.054 | 0.959±0.054 | ＜0.001 | 0.794 |  | 0.779 |  |
| 29 | 9.104±1.168 | 7.268 | 10.882 | 0.916±0.077 | 0.972±0.077 | ＜0.001 | 0.818 |  | 0.804 |  |
| 30 | 9.164±1.226 | 7.583 | 11.139 | 0.926±0.053 | 0.961±0.053 | ＜0.001 | 0.819 |  | 0.804 |  |

TableS5：Eye R

The mean volumes (± SD) of the auto-contours of the Eye-R for each patient, as well as the intersection and union volumes, sensitivity, specificity, the overall Kappa coefficient with its p-value (H₀: κ = 0), and the generalized conformity index (CI-gen) for each case. The overall Kappa coefficient with its p-value (H₀: κ = 0); p < 0.001 indicates agreement significantly higher than random.

| Case | volume | Intersection volume | Union volume | Sensitivity | Specificity | p-value: | Overall kappa: | Mean±SD | CI-gen | Mean±SD |
| --- | --- | --- | --- | --- | --- | --- | --- | --- | --- | --- |
| 1 | 0.305±0.146 | 0.105 | 0.518 | 0.797±0.305 | 0.909±0.305 | ＜0.001 | 0.480 | 0.502±0.072 | 0.468 | 0.485±0.079 |
| 2 | 0.255±0.129 | 0.078 | 0.450 | 0.787±0.325 | 0.896±0.325 | ＜0.001 | 0.435 |  | 0.438 |  |
| 3 | 0.280±0.122 | 0.111 | 0.484 | 0.811±0.207 | 0.842±0.207 | ＜0.001 | 0.404 |  | 0.464 |  |
| 4 | 0.333±0.112 | 0.199 | 0.493 | 0.860±0.134 | 0.906±0.134 | ＜0.001 | 0.578 |  | 0.623 |  |
| 5 | 0.278±0.150 | 0.115 | 0.481 | 0.783±0.284 | 0.909±0.284 | ＜0.001 | 0.475 |  | 0.472 |  |
| 6 | 0.319±0.123 | 0.167 | 0.497 | 0.790±0.216 | 0.940±0.216 | ＜0.001 | 0.576 |  | 0.551 |  |
| 7 | 0.357±0.175 | 0.136 | 0.652 | 0.812±0.259 | 0.880±0.259 | ＜0.001 | 0.436 |  | 0.437 |  |
| 8 | 0.319±0.120 | 0.175 | 0.503 | 0.868±0.179 | 0.892±0.179 | ＜0.001 | 0.562 |  | 0.568 |  |
| 9 | 0.251±0.101 | 0.124 | 0.404 | 0.817±0.212 | 0.949±0.212 | ＜0.001 | 0.603 |  | 0.540 |  |
| 10 | 0.322±0.139 | 0.170 | 0.522 | 0.873±0.172 | 0.817±0.172 | ＜0.001 | 0.438 |  | 0.548 |  |
| 11 | 0.377±0.109 | 0.084 | 0.834 | 0.726±0.327 | 0.986±0.327 | ＜0.001 | 0.457 |  | 0.316 |  |
| 12 | 0.307±0.116 | 0.176 | 0.465 | 0.800±0.212 | 0.926±0.212 | ＜0.001 | 0.551 |  | 0.583 |  |
| 13 | 0.306±0.095 | 0.169 | 0.471 | 0.844±0.184 | 0.901±0.184 | ＜0.001 | 0.549 |  | 0.583 |  |
| 14 | 0.355±0.155 | 0.191 | 0.584 | 0.788±0.167 | 0.917±0.167 | ＜0.001 | 0.528 |  | 0.531 |  |
| 15 | 0.239±0.137 | 0.085 | 0.466 | 0.701±0.310 | 0.921±0.310 | ＜0.001 | 0.393 |  | 0.385 |  |
| 16 | 0.273±0.094 | 0.157 | 0.429 | 0.889±0.150 | 0.891±0.150 | ＜0.001 | 0.582 |  | 0.579 |  |
| 17 | 0.415±0.168 | 0.243 | 0.699 | 0.875±0.105 | 0.918±0.105 | ＜0.001 | 0.593 |  | 0.552 |  |
| 18 | 0.205±0.138 | 0.055 | 0.405 | 0.691±0.364 | 0.900±0.364 | ＜0.001 | 0.335 |  | 0.352 |  |
| 19 | 0.362±0.153 | 0.222 | 0.579 | 0.961±0.078 | 0.878±0.078 | ＜0.001 | 0.602 |  | 0.570 |  |
| 20 | 0.417±0.106 | 0.143 | 0.804 | 0.777±0.295 | 0.988±0.295 | ＜0.001 | 0.563 |  | 0.413 |  |
| 21 | 0.443±0.160 | 0.169 | 0.948 | 0.801±0.128 | 0.989±0.128 | ＜0.001 | 0.530 |  | 0.375 |  |
| 22 | 0.371±0.187 | 0.137 | 0.733 | 0.816±0.247 | 0.878±0.247 | ＜0.001 | 0.403 |  | 0.398 |  |
| 23 | 0.316±0.040 | 0.122 | 0.602 | 0.760±0.222 | 0.984±0.222 | ＜0.001 | 0.566 |  | 0.423 |  |
| 24 | 0.363±0.154 | 0.168 | 0.631 | 0.868±0.134 | 0.863±0.134 | ＜0.001 | 0.478 |  | 0.482 |  |
| 25 | 0.299±0.157 | 0.115 | 0.539 | 0.804±0.270 | 0.906±0.270 | ＜0.001 | 0.472 |  | 0.452 |  |
| 26 | 0.356±0.164 | 0.145 | 0.570 | 0.840±0.266 | 0.838±0.266 | ＜0.001 | 0.433 |  | 0.511 |  |
| 27 | 0.270±0.102 | 0.118 | 0.430 | 0.746±0.261 | 0.948±0.261 | ＜0.001 | 0.523 |  | 0.507 |  |
| 28 | 0.316±0.129 | 0.147 | 0.513 | 0.854±0.248 | 0.869±0.248 | ＜0.001 | 0.474 |  | 0.540 |  |
| 29 | 0.364±0.160 | 0.146 | 0.724 | 0.775±0.245 | 0.990±0.245 | ＜0.001 | 0.561 |  | 0.406 |  |
| 30 | 0.310±0.138 | 0.145 | 0.549 | 0.857±0.198 | 0.876±0.198 | ＜0.001 | 0.484 |  | 0.475 |  |

TableS6：Len L

The mean volumes (± SD) of the auto-contours of the Len-L for each patient, as well as the intersection and union volumes, sensitivity, specificity, the overall Kappa coefficient with its p-value (H₀: κ = 0), and the generalized conformity index (CI-gen) for each case. The overall Kappa coefficient with its p-value (H₀: κ = 0); p < 0.001 indicates agreement significantly higher than random.

| Case | volume | Intersection volume | Union volume | Sensitivity | Specificity | p-value: | Overall kappa: | Mean±SD | CI-gen | Mean±SD |
| --- | --- | --- | --- | --- | --- | --- | --- | --- | --- | --- |
| 1 | 0.307±0.124 | 0.136 | 0.492 | 0.810±0.223 | 0.863±0.223 | ＜0.001 | 0.456 | 0.485±0.115 | 0.367 | 0.502±0.064 |
| 2 | 0.265±0.108 | 0.114 | 0.470 | 0.784±0.235 | 0.874±0.235 | ＜0.001 | 0.418 |  | 0.461 |  |
| 3 | 0.349±0.138 | 0.156 | 0.626 | 0.815±0.122 | 0.885±0.122 | ＜0.001 | 0.469 |  | 0.463 |  |
| 4 | 0.318±0.129 | 0.158 | 0.517 | 0.754±0.284 | 0.947±0.284 | ＜0.001 | 0.543 |  | 0.510 |  |
| 5 | 0.258±0.085 | 0.162 | 0.377 | 0.857±0.171 | 0.930±0.171 | ＜0.001 | 0.648 |  | 0.624 |  |
| 6 | 0.312±0.111 | 0.141 | 0.518 | 0.764±0.197 | 0.950±0.197 | ＜0.001 | 0.556 |  | 0.500 |  |
| 7 | 0.344±0.209 | 0.130 | 0.620 | 0.731±0.313 | 0.866±0.313 | ＜0.001 | 0.368 |  | 0.417 |  |
| 8 | 0.310±0.108 | 0.175 | 0.513 | 0.892±0.133 | 0.893±0.133 | ＜0.001 | 0.563 |  | 0.557 |  |
| 9 | 0.228±0.127 | 0.078 | 0.404 | 0.733±0.298 | 0.920±0.298 | ＜0.001 | 0.433 |  | 0.435 |  |
| 10 | 0.259±0.078 | 0.149 | 0.405 | 0.912±0.071 | 0.816±0.071 | ＜0.001 | 0.499 |  | 0.577 |  |
| 11 | 0.322±0.104 | 0.169 | 0.570 | 0.882±0.115 | 0.893±0.115 | ＜0.001 | 0.527 |  | 0.512 |  |
| 12 | 0.302±0.064 | 0.165 | 0.465 | 0.843±0.102 | 0.936±0.102 | ＜0.001 | 0.619 |  | 0.581 |  |
| 13 | 0.346±0.160 | 0.195 | 0.615 | 0.909±0.113 | 0.869±0.113 | ＜0.001 | 0.512 |  | 0.523 |  |
| 14 | 0.379±0.118 | 0.217 | 0.568 | 0.870±0.144 | 0.897±0.144 | ＜0.001 | 0.589 |  | 0.598 |  |
| 15 | 0.282±0.165 | 0.095 | 0.508 | 0.744±0.297 | 0.855±0.297 | ＜0.001 | 0.347 |  | 0.417 |  |
| 16 | 0.292±0.093 | 0.131 | 0.450 | 0.858±0.170 | 0.847±0.170 | ＜0.001 | 0.461 |  | 0.552 |  |
| 17 | 0.359±0.139 | 0.185 | 0.614 | 0.863±0.180 | 0.876±0.180 | ＜0.001 | 0.503 |  | 0.517 |  |
| 18 | 0.238±0.209 | 0.080 | 0.395 | 0.648±0.439 | 0.832±0.439 | ＜0.001 | 0.020 |  | 0.456 |  |
| 19 | 0.349±0.198 | 0.145 | 0.651 | 0.763±0.182 | 0.899±0.182 | ＜0.001 | 0.435 |  | 0.434 |  |
| 20 | 0.310±0.142 | 0.122 | 0.566 | 0.810±0.167 | 0.897±0.167 | ＜0.001 | 0.460 |  | 0.450 |  |
| 21 | 0.375±0.177 | 0.225 | 0.671 | 0.921±0.078 | 0.894±0.078 | ＜0.001 | 0.559 |  | 0.523 |  |
| 22 | 0.274±0.110 | 0.153 | 0.448 | 0.878±0.147 | 0.914±0.147 | ＜0.001 | 0.587 |  | 0.564 |  |
| 23 | 0.241±0.090 | 0.132 | 0.426 | 0.882±0.119 | 0.901±0.119 | ＜0.001 | 0.548 |  | 0.515 |  |
| 24 | 0.328±0.092 | 0.209 | 0.540 | 0.930±0.045 | 0.909±0.045 | ＜0.001 | 0.624 |  | 0.583 |  |
| 25 | 0.358±0.181 | 0.173 | 0.628 | 0.871±0.180 | 0.831±0.180 | ＜0.001 | 0.429 |  | 0.495 |  |
| 26 | 0.375±0.120 | 0.218 | 0.555 | 0.899±0.110 | 0.808±0.110 | ＜0.001 | 0.498 |  | 0.600 |  |
| 27 | 0.288±0.122 | 0.123 | 0.543 | 0.837±0.173 | 0.865±0.173 | ＜0.001 | 0.417 |  | 0.448 |  |
| 28 | 0.324±0.134 | 0.137 | 0.553 | 0.810±0.235 | 0.889±0.235 | ＜0.001 | 0.479 |  | 0.480 |  |
| 29 | 0.352±0.211 | 0.149 | 0.664 | 0.799±0.194 | 0.932±0.194 | ＜0.001 | 0.502 |  | 0.445 |  |
| 30 | 0.311±0.153 | 0.145 | 0.544 | 0.779±0.262 | 0.899±0.262 | ＜0.001 | 0.475 |  | 0.469 |  |

TableS7：Len R

The mean volumes (± SD) of the auto-contours of the Len-R for each patient, as well as the intersection and union volumes, sensitivity, specificity, the overall Kappa coefficient with its p-value (H₀: κ = 0), and the generalized conformity index (CI-gen) for each case. The overall Kappa coefficient with its p-value (H₀: κ = 0); p < 0.001 indicates agreement significantly higher than random.

| Case | volume | Intersection volume | Union volume | Sensitivity | Specificity | p-value: | Overall kappa: | Mean±SD | CI-gen | Mean±SD |
| --- | --- | --- | --- | --- | --- | --- | --- | --- | --- | --- |
| 1 | 67.954±10.211 | 52.037 | 85.067 | 0.892±0.097 | 0.996±0.004 | ＜0.001 | 0.855 | 0.866±0.015 | 0.763 | 0.779±0.024 |
| 2 | 52.711±7.922 | 41.689 | 64.515 | 0.908±0.094 | 0.997±0.004 | ＜0.001 | 0.872 |  | 0.788 |  |
| 3 | 81.401±11.039 | 65.768 | 98.313 | 0.913±0.086 | 0.996±0.005 | ＜0.001 | 0.879 |  | 0.802 |  |
| 4 | 69.532±10.877 | 54.253 | 86.026 | 0.901±0.102 | 0.997±0.003 | ＜0.001 | 0.866 |  | 0.777 |  |
| 5 | 88.946±13.401 | 68.791 | 109.640 | 0.903±0.099 | 0.996±0.004 | ＜0.001 | 0.864 |  | 0.777 |  |
| 6 | 89.794±10.443 | 73.155 | 107.413 | 0.913±0.075 | 0.997±0.003 | ＜0.001 | 0.885 |  | 0.809 |  |
| 7 | 57.673±9.296 | 41.741 | 73.116 | 0.873±0.108 | 0.997±0.003 | ＜0.001 | 0.840 |  | 0.738 |  |
| 8 | 65.421±9.569 | 51.321 | 80.897 | 0.906±0.092 | 0.997±0.004 | ＜0.001 | 0.867 |  | 0.780 |  |
| 9 | 94.784±13.965 | 71.290 | 117.589 | 0.886±0.097 | 0.997±0.004 | ＜0.001 | 0.854 |  | 0.762 |  |
| 10 | 74.673±10.256 | 57.600 | 91.922 | 0.893±0.092 | 0.997±0.004 | ＜0.001 | 0.861 |  | 0.774 |  |
| 11 | 55.611±8.688 | 41.247 | 70.637 | 0.881±0.099 | 0.997±0.003 | ＜0.001 | 0.845 |  | 0.745 |  |
| 12 | 77.121±9.902 | 60.377 | 93.824 | 0.900±0.085 | 0.997±0.004 | ＜0.001 | 0.869 |  | 0.786 |  |
| 13 | 87.416±12.895 | 69.277 | 107.158 | 0.908±0.095 | 0.996±0.004 | ＜0.001 | 0.871 |  | 0.787 |  |
| 14 | 71.081±9.210 | 55.479 | 86.568 | 0.897±0.088 | 0.997±0.004 | ＜0.001 | 0.867 |  | 0.783 |  |
| 15 | 96.169±11.593 | 78.492 | 114.342 | 0.912±0.080 | 0.996±0.004 | ＜0.001 | 0.885 |  | 0.812 |  |
| 16 | 74.694±10.345 | 57.077 | 91.616 | 0.890±0.092 | 0.997±0.003 | ＜0.001 | 0.862 |  | 0.744 |  |
| 17 | 85.523±12.616 | 67.337 | 103.833 | 0.902±0.099 | 0.997±0.004 | ＜0.001 | 0.872 |  | 0.789 |  |
| 18 | 79.359±10.997 | 62.874 | 97.576 | 0.908±0.084 | 0.997±0.004 | ＜0.001 | 0.871 |  | 0.786 |  |
| 19 | 66.901±9.129 | 51.076 | 83.827 | 0.893±0.093 | 0.997±0.003 | ＜0.001 | 0.856 |  | 0.762 |  |
| 20 | 75.735±10.714 | 58.592 | 92.529 | 0.899±0.095 | 0.996±0.004 | ＜0.001 | 0.865 |  | 0.780 |  |
| 21 | 64.245±9.258 | 50.403 | 78.590 | 0.908±0.092 | 0.997±0.003 | ＜0.001 | 0.873 |  | 0.797 |  |
| 22 | 80.279±11.010 | 61.236 | 97.233 | 0.898±0.099 | 0.996±0.004 | ＜0.001 | 0.865 |  | 0.782 |  |
| 23 | 58.806±10.825 | 41.439 | 76.858 | 0.869±0.121 | 0.996±0.004 | ＜0.001 | 0.823 |  | 0.715 |  |
| 24 | 67.837±9.853 | 50.989 | 84.680 | 0.889±0.097 | 0.997±0.004 | ＜0.001 | 0.853 |  | 0.759 |  |
| 25 | 59.728±7.469 | 45.700 | 74.578 | 0.892±0.086 | 0.997±0.003 | ＜0.001 | 0.857 |  | 0.764 |  |
| 26 | 95.187±12.262 | 77.723 | 113.301 | 0.916±0.085 | 0.996±0.005 | ＜0.001 | 0.885 |  | 0.813 |  |
| 27 | 85.833±11.804 | 68.032 | 104.538 | 0.912±0.087 | 0.996±0.005 | ＜0.001 | 0.873 |  | 0.793 |  |
| 28 | 72.255±10.741 | 58.569 | 85.799 | 0.934±0.093 | 0.995±0.005 | ＜0.001 | 0.865 |  | 0.777 |  |
| 29 | 59.200±7.946 | 48.173 | 71.564 | 0.915±0.082 | 0.997±0.004 | ＜0.001 | 0.884 |  | 0.804 |  |
| 30 | 63.145±7.611 | 52.023 | 75.160 | 0.924±0.074 | 0.998±0.003 | ＜0.001 | 0.894 |  | 0.818 |  |

TableS8：Mandible

The mean volumes (± SD) of the auto-contours of the Mandible for each patient, as well as the intersection and union volumes, sensitivity, specificity, the overall Kappa coefficient with its p-value (H₀: κ = 0), and the generalized conformity index (CI-gen) for each case. The overall Kappa coefficient with its p-value (H₀: κ = 0); p < 0.001 indicates agreement significantly higher than random.

| Case | volume | Intersection volume | Union volume | Sensitivity | Specificity | p-value: | Overall kappa: | Mean±SD | CI-gen | Mean±SD |
| --- | --- | --- | --- | --- | --- | --- | --- | --- | --- | --- |
| 1 | 0.575±0.160 | 0.251 | 0.999 | 0.801±0.145 | 0.977±0.145 | ＜0.001 | 0.614 | 0.621±0.071 | 0.488 | 0.508±0.073 |
| 2 | 0.407±0.105 | 0.155 | 0.693 | 0.767±0.170 | 0.986±0.170 | ＜0.001 | 0.616 |  | 0.479 |  |
| 3 | 0.725±0.225 | 0.358 | 1.272 | 0.786±0.169 | 0.979±0.169 | ＜0.001 | 0.618 |  | 0.488 |  |
| 4 | 0.557±0.154 | 0.298 | 0.873 | 0.823±0.183 | 0.984±0.183 | ＜0.001 | 0.689 |  | 0.540 |  |
| 5 | 0.523±0.058 | 0.288 | 0.847 | 0.800±0.054 | 0.975±0.054 | ＜0.001 | 0.657 |  | 0.546 |  |
| 6 | 0.675±0.124 | 0.371 | 1.025 | 0.822±0.091 | 0.966±0.091 | ＜0.001 | 0.665 |  | 0.584 |  |
| 7 | 0.555±0.048 | 0.375 | 0.772 | 0.847±0.036 | 0.987±0.036 | ＜0.001 | 0.768 |  | 0.668 |  |
| 8 | 0.492±0.085 | 0.201 | 1.053 | 0.789±0.148 | 0.990±0.148 | ＜0.001 | 0.533 |  | 0.376 |  |
| 9 | 0.407±0.075 | 0.207 | 0.674 | 0.724±0.147 | 0.981±0.147 | ＜0.001 | 0.616 |  | 0.500 |  |
| 10 | 0.259±0.078 | 0.149 | 0.405 | 0.912±0.071 | 0.816±0.071 | ＜0.001 | 0.499 |  | 0.455 |  |
| 11 | 0.524±0.175 | 0.216 | 0.918 | 0.756±0.202 | 0.978±0.202 | ＜0.001 | 0.588 |  | 0.465 |  |
| 12 | 0.681±0.195 | 0.326 | 1.121 | 0.743±0.208 | 0.990±0.208 | ＜0.001 | 0.642 |  | 0.503 |  |
| 13 | 0.517±0.084 | 0.307 | 0.799 | 0.832±0.048 | 0.983±0.048 | ＜0.001 | 0.708 |  | 0.591 |  |
| 14 | 0.549±0.181 | 0.196 | 1.003 | 0.753±0.184 | 0.976±0.184 | ＜0.001 | 0.560 |  | 0.436 |  |
| 15 | 0.466±0.136 | 0.238 | 0.921 | 0.896±0.078 | 0.960±0.078 | ＜0.001 | 0.571 |  | 0.447 |  |
| 16 | 0.592±0.145 | 0.382 | 0.873 | 0.861±0.097 | 0.972±0.097 | ＜0.001 | 0.723 |  | 0.633 |  |
| 17 | 0.437±0.127 | 0.212 | 0.751 | 0.779±0.155 | 0.974±0.155 | ＜0.001 | 0.611 |  | 0.493 |  |
| 18 | 0.456±0.041 | 0.215 | 0.700 | 0.785±0.067 | 0.967±0.067 | ＜0.001 | 0.625 |  | 0.551 |  |
| 19 | 0.658±0.173 | 0.310 | 1.158 | 0.774±0.116 | 0.971±0.116 | ＜0.001 | 0.595 |  | 0.480 |  |
| 20 | 0.495±0.157 | 0.191 | 0.905 | 0.753±0.174 | 0.973±0.174 | ＜0.001 | 0.560 |  | 0.440 |  |
| 21 | 0.530±0.142 | 0.210 | 0.937 | 0.779±0.226 | 0.966±0.226 | ＜0.001 | 0.567 |  | 0.463 |  |
| 22 | 0.561±0.102 | 0.290 | 0.860 | 0.779±0.183 | 0.970±0.183 | ＜0.001 | 0.634 |  | 0.557 |  |
| 23 | 0.513±0.121 | 0.191 | 0.876 | 0.734±0.134 | 0.994±0.134 | ＜0.001 | 0.621 |  | 0.467 |  |
| 24 | 0.493±0.148 | 0.163 | 0.972 | 0.734±0.116 | 0.959±0.116 | ＜0.001 | 0.490 |  | 0.387 |  |
| 25 | 0.522±0.122 | 0.303 | 0.842 | 0.844±0.097 | 0.980±0.097 | ＜0.001 | 0.690 |  | 0.567 |  |
| 26 | 0.612±0.099 | 0.409 | 0.917 | 0.868±0.079 | 0.980±0.079 | ＜0.001 | 0.740 |  | 0.631 |  |
| 27 | 0.603±0.147 | 0.246 | 1.106 | 0.790±0.111 | 0.977±0.111 | ＜0.001 | 0.585 |  | 0.454 |  |
| 28 | 0.372±0.087 | 0.112 | 0.665 | 0.695±0.220 | 0.961±0.220 | ＜0.001 | 0.486 |  | 0.415 |  |
| 29 | 0.618±0.112 | 0.380 | 0.980 | 0.874±0.082 | 0.964±0.082 | ＜0.001 | 0.687 |  | 0.588 |  |
| 30 | 0.612±0.172 | 0.321 | 1.011 | 0.830±0.166 | 0.976±0.166 | ＜0.001 | 0.658 |  | 0.537 |  |

TableS9：Optic Nerves L

The mean volumes (± SD) of the auto-contours of the Optic Nerves-L for each patient, as well as the intersection and union volumes, sensitivity, specificity, the overall Kappa coefficient with its p-value (H₀: κ = 0), and the generalized conformity index (CI-gen) for each case. The overall Kappa coefficient with its p-value (H₀: κ = 0); p < 0.001 indicates agreement significantly higher than random.

TableS10：Optic Nerves R

| Case | volume | Intersection volume | Union volume | Sensitivity | Specificity | p-value: | Overall kappa: | Mean±SD | CI-gen | Mean±SD |
| --- | --- | --- | --- | --- | --- | --- | --- | --- | --- | --- |
| 1 | 0.652±0.249 | 0.235 | 1.292 | 0.764±0.088 | 0.971±0.088 | ＜0.001 | 0.528 | 0.599±0.075 | 0.403 | 0.480±0.077 |
| 2 | 0.628±0.179 | 0.274 | 1.090 | 0.803±0.121 | 0.978±0.121 | ＜0.001 | 0.617 |  | 0.489 |  |
| 3 | 0.817±0.310 | 0.378 | 1.600 | 0.838±0.149 | 0.966±0.149 | ＜0.001 | 0.563 |  | 0.437 |  |
| 4 | 0.592±0.166 | 0.294 | 1.014 | 0.798±0.151 | 0.979±0.151 | ＜0.001 | 0.632 |  | 0.505 |  |
| 5 | 0.660±0.267 | 0.314 | 1.224 | 0.761±0.194 | 0.979±0.194 | ＜0.001 | 0.584 |  | 0.451 |  |
| 6 | 0.685±0.049 | 0.434 | 1.062 | 0.840±0.011 | 0.987±0.011 | ＜0.001 | 0.725 |  | 0.599 |  |
| 7 | 0.484±0.187 | 0.203 | 0.954 | 0.808±0.111 | 0.975±0.111 | ＜0.001 | 0.557 |  | 0.421 |  |
| 8 | 0.626±0.202 | 0.328 | 1.069 | 0.858±0.165 | 0.933±0.165 | ＜0.001 | 0.589 |  | 0.519 |  |
| 9 | 0.455±0.131 | 0.192 | 0.809 | 0.724±0.220 | 0.963±0.220 | ＜0.001 | 0.536 |  | 0.444 |  |
| 10 | 0.656±0.190 | 0.346 | 1.160 | 0.859±0.098 | 0.967±0.098 | ＜0.001 | 0.627 |  | 0.509 |  |
| 11 | 0.484±0.186 | 0.185 | 0.876 | 0.727±0.275 | 0.965±0.275 | ＜0.001 | 0.529 |  | 0.436 |  |
| 12 | 0.752±0.363 | 0.202 | 1.499 | 0.744±0.206 | 0.973±0.206 | ＜0.001 | 0.500 |  | 0.377 |  |
| 13 | 0.676±0.138 | 0.261 | 1.475 | 0.727±0.091 | 0.999±0.091 | ＜0.001 | 0.519 |  | 0.353 |  |
| 14 | 0.680±0.288 | 0.191 | 1.473 | 0.700±0.170 | 0.971±0.170 | ＜0.001 | 0.448 |  | 0.329 |  |
| 15 | 0.565±0.195 | 0.249 | 1.111 | 0.794±0.098 | 0.961±0.098 | ＜0.001 | 0.537 |  | 0.420 |  |
| 16 | 0.650±0.102 | 0.397 | 1.046 | 0.849±0.052 | 0.980±0.052 | ＜0.001 | 0.699 |  | 0.576 |  |
| 17 | 0.501±0.160 | 0.243 | 0.868 | 0.795±0.112 | 0.977±0.112 | ＜0.001 | 0.623 |  | 0.498 |  |
| 18 | 0.608±0.085 | 0.395 | 0.910 | 0.891±0.079 | 0.976±0.079 | ＜0.001 | 0.739 |  | 0.635 |  |
| 19 | 0.804±0.307 | 0.486 | 1.385 | 0.886±0.079 | 0.967±0.079 | ＜0.001 | 0.663 |  | 0.546 |  |
| 20 | 0.548±0.227 | 0.270 | 1.048 | 0.840±0.133 | 0.966±0.133 | ＜0.001 | 0.580 |  | 0.456 |  |
| 21 | 0.553±0.134 | 0.287 | 0.855 | 0.806±0.110 | 0.980±0.110 | ＜0.001 | 0.676 |  | 0.565 |  |
| 22 | 0.555±0.280 | 0.190 | 1.150 | 0.737±0.188 | 0.961±0.188 | ＜0.001 | 0.475 |  | 0.367 |  |
| 23 | 0.556±0.113 | 0.294 | 0.891 | 0.836±0.082 | 0.983±0.082 | ＜0.001 | 0.684 |  | 0.558 |  |
| 24 | 0.575±0.090 | 0.336 | 0.891 | 0.850±0.069 | 0.978±0.069 | ＜0.001 | 0.703 |  | 0.592 |  |
| 25 | 0.570±0.114 | 0.319 | 1.041 | 0.831±0.052 | 0.982±0.052 | ＜0.001 | 0.635 |  | 0.493 |  |
| 26 | 0.632±0.093 | 0.352 | 1.011 | 0.845±0.058 | 0.978±0.058 | ＜0.001 | 0.687 |  | 0.569 |  |
| 27 | 0.677±0.333 | 0.266 | 1.296 | 0.790±0.170 | 0.971±0.170 | ＜0.001 | 0.555 |  | 0.431 |  |
| 28 | 0.376±0.130 | 0.157 | 0.650 | 0.807±0.198 | 0.956±0.198 | ＜0.001 | 0.576 |  | 0.485 |  |
| 29 | 0.644±0.249 | 0.288 | 1.172 | 0.822±0.201 | 0.969±0.201 | ＜0.001 | 0.592 |  | 0.470 |  |
| 30 | 0.578±0.246 | 0.295 | 1.125 | 0.880±0.130 | 0.962±0.130 | ＜0.001 | 0.579 |  | 0.456 |  |

The mean volumes (± SD) of the auto-contours of the Optic Nerves-R for each patient, as well as the intersection and union volumes, sensitivity, specificity, the overall Kappa coefficient with its p-value (H₀: κ = 0), and the generalized conformity index (CI-gen) for each case. The overall Kappa coefficient with its p-value (H₀: κ = 0); p < 0.001 indicates agreement significantly higher than random.

| Case | volume | Intersection volume | Union volume | Sensitivity | Specificity | p-value: | Overall kappa: | Mean±SD | CI-gen | Mean±SD |
| --- | --- | --- | --- | --- | --- | --- | --- | --- | --- | --- |
| 1 | 0.328±0.089 | 0.117 | 0.613 | 0.731±0.195 | 0.899±0.090 | ＜0.001 | 0.401 | 0.370±0.115 | 0.416 | 0.394±0.098 |
| 2 | 0.410±0.155 | 0.164 | 0.822 | 0.836±0.148 | 0.873±0.112 | ＜0.001 | 0.407 |  | 0.413 |  |
| 3 | 0.301±0.012 | 0.131 | 0.528 | 0.786±0.173 | 0.943±0.063 | ＜0.001 | 0.542 |  | 0.478 |  |
| 4 | 0.468±0.180 | 0.202 | 0.801 | 0.794±0.232 | 0.928±0.080 | ＜0.001 | 0.522 |  | 0.488 |  |
| 5 | 0.345±0.115 | 0.115 | 0.565 | 0.731±0.237 | 0.918±0.080 | ＜0.001 | 0.430 |  | 0.469 |  |
| 6 | 0.395±0.071 | 0.203 | 0.714 | 0.733±0.211 | 0.896±0.172 | ＜0.001 | 0.430 |  | 0.466 |  |
| 7 | 0.189±0.078 | 0.026 | 0.423 | 0.628±0.355 | 0.931±0.048 | ＜0.001 | 0.296 |  | 0.273 |  |
| 8 | 0.262±0.065 | 0.166 | 0.399 | 0.979±0.027 | 0.806±0.138 | ＜0.001 | 0.550 |  | 0.586 |  |
| 9 | 0.498±0.081 | 0.202 | 0.825 | 0.786±0.188 | 0.882±0.030 | ＜0.001 | 0.445 |  | 0.494 |  |
| 10 | 0.116±0.084 | 0.000 | 0.301 | 0.574±0.418 | 0.931±0.052 | ＜0.001 | 0.198 |  | 0.179 |  |
| 11 | 0.255±0.076 | 0.082 | 0.497 | 0.790±0.108 | 0.891±0.095 | ＜0.001 | 0.410 |  | 0.401 |  |
| 12 | 0.453±0.139 | 0.159 | 0.763 | 0.745±0.253 | 0.825±0.144 | ＜0.001 | 0.368 |  | 0.429 |  |
| 13 | 0.252±0.104 | 0.026 | 0.543 | 0.731±0.225 | 0.883±0.111 | ＜0.001 | 0.294 |  | 0.297 |  |
| 14 | 0.126±0.056 | 0.000 | 0.254 | 0.641±0.440 | 0.944±0.037 | ＜0.001 | 0.310 |  | 0.295 |  |
| 15 | 0.259±0.083 | 0.146 | 0.397 | 0.838±0.126 | 0.925±0.104 | ＜0.001 | 0.600 |  | 0.584 |  |
| 16 | 0.294±0.149 | 0.101 | 0.508 | 0.655±0.304 | 0.942±0.071 | ＜0.001 | 0.381 |  | 0.414 |  |
| 17 | 0.208±0.177 | 0.000 | 0.515 | 0.446±0.308 | 0.835±0.215 | ＜0.001 | 0.099 |  | 0.175 |  |
| 18 | 0.397±0.160 | 0.119 | 0.729 | 0.670±0.253 | 0.893±0.063 | ＜0.001 | 0.344 |  | 0.383 |  |
| 19 | 0.372±0.202 | 0.074 | 0.725 | 0.714±0.270 | 0.922±0.095 | ＜0.001 | 0.383 |  | 0.364 |  |
| 20 | 0.380±0.174 | 0.111 | 0.755 | 0.654±0.240 | 0.887±0.132 | ＜0.001 | 0.302 |  | 0.344 |  |
| 21 | 0.319±0.058 | 0.114 | 0.643 | 0.711±0.212 | 0.915±0.056 | ＜0.001 | 0.401 |  | 0.377 |  |
| 22 | 0.299±0.106 | 0.077 | 0.575 | 0.710±0.153 | 0.917±0.071 | ＜0.001 | 0.397 |  | 0.379 |  |
| 23 | 0.406±0.135 | 0.142 | 0.849 | 0.769±0.135 | 0.917±0.075 | ＜0.001 | 0.419 |  | 0.373 |  |
| 24 | 0.389±0.253 | 0.064 | 0.759 | 0.647±0.380 | 0.912±0.091 | ＜0.001 | 0.302 |  | 0.329 |  |
| 25 | 0.267±0.101 | 0.102 | 0.575 | 0.768±0.177 | 0.861±0.122 | ＜0.001 | 0.332 |  | 0.358 |  |
| 26 | 0.348±0.182 | 0.105 | 0.648 | 0.777±0.271 | 0.892±0.130 | ＜0.001 | 0.403 |  | 0.415 |  |
| 27 | 0.152±0.058 | 0.047 | 0.316 | 0.863±0.177 | 0.809±0.170 | ＜0.001 | 0.291 |  | 0.340 |  |
| 28 | 0.288±0.062 | 0.155 | 0.439 | 0.847±0.178 | 0.864±0.052 | ＜0.001 | 0.463 |  | 0.505 |  |
| 29 | 0.235±0.068 | 0.071 | 0.376 | 0.739±0.235 | 0.790±0.137 | ＜0.001 | 0.244 |  | 0.472 |  |
| 30 | 0.226±0.202 | 0.000 | 0.525 | 0.582±0.451 | 0.869±0.152 | ＜0.001 | 0.123 |  | 0.329 |  |

TableS11：Pituitary Gland

The mean volumes (± SD) of the auto-contours of the Pituitary Gland for each patient, as well as the intersection and union volumes, sensitivity, specificity, the overall Kappa coefficient with its p-value (H₀: κ = 0), and the generalized conformity index (CI-gen) for each case. The overall Kappa coefficient with its p-value (H₀: κ = 0); p < 0.001 indicates agreement significantly higher than random.

| Case | volume | Intersection volume | Union volume | Sensitivity | Specificity | p-value: | Overall kappa: | Mean±SD | CI-gen | Mean±SD |
| --- | --- | --- | --- | --- | --- | --- | --- | --- | --- | --- |
| 1 | 22.288±3.635 | 15.381 | 29.783 | 0.878±0.106 | 0.982±0.106 | ＜0.001 | 0.784 | 0.743±0.086 | 0.702 | 0.636±0.113 |
| 2 | 30.889±4.289 | 22.977 | 39.979 | 0.897±0.077 | 0.984±0.077 | ＜0.001 | 0.816 |  | 0.740 |  |
| 3 | 30.786±3.073 | 22.100 | 41.526 | 0.895±0.055 | 0.988±0.055 | ＜0.001 | 0.808 |  | 0.711 |  |
| 4 | 23.109±3.365 | 16.977 | 30.376 | 0.891±0.099 | 0.996±0.099 | ＜0.001 | 0.835 |  | 0.729 |  |
| 5 | 18.084±6.834 | 8.770 | 35.614 | 0.829±0.102 | 0.991±0.102 | ＜0.001 | 0.599 |  | 0.441 |  |
| 6 | 52.918±9.913 | 32.100 | 67.374 | 0.865±0.173 | 0.981±0.173 | ＜0.001 | 0.756 |  | 0.700 |  |
| 7 | 30.194±4.653 | 22.348 | 40.285 | 0.900±0.074 | 0.981±0.074 | ＜0.001 | 0.803 |  | 0.722 |  |
| 8 | 35.670±3.984 | 26.797 | 46.065 | 0.894±0.072 | 0.988±0.072 | ＜0.001 | 0.823 |  | 0.742 |  |
| 9 | 8.419±4.142 | 3.628 | 15.851 | 0.795±0.110 | 0.950±0.110 | ＜0.001 | 0.536 |  | 0.446 |  |
| 10 | 39.592±15.589 | 24.171 | 69.395 | 0.875±0.092 | 0.989±0.092 | ＜0.001 | 0.686 |  | 0.539 |  |
| 11 | 27.236±2.272 | 19.279 | 36.237 | 0.888±0.052 | 0.985±0.052 | ＜0.001 | 0.800 |  | 0.713 |  |
| 12 | 41.345±16.624 | 26.780 | 70.874 | 0.912±0.081 | 0.987±0.081 | ＜0.001 | 0.709 |  | 0.569 |  |
| 13 | 29.172±4.808 | 20.611 | 39.050 | 0.876±0.106 | 0.985±0.106 | ＜0.001 | 0.792 |  | 0.705 |  |
| 14 | 25.486±3.943 | 18.248 | 34.806 | 0.895±0.091 | 0.979±0.091 | ＜0.001 | 0.786 |  | 0.701 |  |
| 15 | 26.169±13.671 | 14.055 | 50.028 | 0.873±0.099 | 0.990±0.099 | ＜0.001 | 0.633 |  | 0.477 |  |
| 16 | 37.354±4.669 | 28.779 | 47.689 | 0.901±0.079 | 0.984±0.079 | ＜0.001 | 0.826 |  | 0.755 |  |
| 17 | 32.900±4.530 | 24.485 | 42.249 | 0.893±0.098 | 0.985±0.098 | ＜0.001 | 0.818 |  | 0.742 |  |
| 18 | 21.443±6.563 | 12.150 | 39.196 | 0.875±0.058 | 0.989±0.058 | ＜0.001 | 0.658 |  | 0.507 |  |
| 19 | 45.532±23.470 | 26.160 | 85.272 | 0.890±0.097 | 0.985±0.097 | ＜0.001 | 0.646 |  | 0.499 |  |
| 20 | 16.857±5.197 | 8.880 | 30.137 | 0.845±0.105 | 0.991±0.105 | ＜0.001 | 0.657 |  | 0.505 |  |
| 21 | 37.500±4.954 | 26.398 | 49.176 | 0.871±0.093 | 0.989±0.093 | ＜0.001 | 0.804 |  | 0.714 |  |
| 22 | 34.162±4.234 | 26.460 | 42.843 | 0.907±0.075 | 0.988±0.075 | ＜0.001 | 0.841 |  | 0.769 |  |
| 23 | 18.330±6.740 | 9.941 | 33.158 | 0.846±0.103 | 0.988±0.103 | ＜0.001 | 0.649 |  | 0.500 |  |
| 24 | 19.873±2.840 | 13.059 | 28.313 | 0.854±0.086 | 0.997±0.086 | ＜0.001 | 0.784 |  | 0.655 |  |
| 25 | 24.348±4.270 | 17.091 | 32.027 | 0.875±0.126 | 0.990±0.126 | ＜0.001 | 0.805 |  | 0.713 |  |
| 26 | 22.741±8.058 | 11.823 | 41.959 | 0.836±0.109 | 0.989±0.109 | ＜0.001 | 0.636 |  | 0.484 |  |
| 27 | 17.691±2.962 | 12.498 | 23.694 | 0.881±0.101 | 0.982±0.101 | ＜0.001 | 0.788 |  | 0.706 |  |
| 28 | 11.580±3.403 | 6.797 | 18.396 | 0.844±0.096 | 0.972±0.096 | ＜0.001 | 0.682 |  | 0.577 |  |
| 29 | 37.833±3.419 | 29.895 | 46.973 | 0.913±0.054 | 0.987±0.054 | ＜0.001 | 0.848 |  | 0.781 |  |
| 30 | 38.660±17.696 | 24.077 | 70.364 | 0.914±0.071 | 0.988±0.071 | ＜0.001 | 0.677 |  | 0.529 |  |

TableS12：Parotid Gland L

The mean volumes (± SD) of the auto-contours of the Parotid Gland-L for each patient, as well as the intersection and union volumes, sensitivity, specificity, the overall Kappa coefficient with its p-value (H₀: κ = 0), and the generalized conformity index (CI-gen) for each case. The overall Kappa coefficient with its p-value (H₀: κ = 0); p < 0.001 indicates agreement significantly higher than random.

TableS13：Parotid Gland R

The mean volumes (± SD) of the auto-contours of the Parotid Gland-R for each patient, as well as the intersection and union volumes, sensitivity, specificity, the overall Kappa coefficient with its p-value (H₀: κ = 0), and the generalized conformity index (CI-gen) for each case. The overall Kappa coefficient with its p-value (H₀: κ = 0); p < 0.001 indicates agreement significantly higher than random.

| Case | volume | Intersection volume | Union volume | Sensitivity | Specificity | p-value: | Overall kappa: | Mean±SD | CI-gen | Mean±SD |
| --- | --- | --- | --- | --- | --- | --- | --- | --- | --- | --- |
| 1 | 23.288±1.531 | 16.897 | 30.139 | 0.894±0.076 | 0.984±0.076 | ＜0.001 | 0.809 | 0.788±0.076 | 0.735 | 0.705±0.088 |
| 2 | 30.905±3.668 | 23.938 | 39.209 | 0.904±0.082 | 0.987±0.082 | ＜0.001 | 0.835 |  | 0.761 |  |
| 3 | 31.069±2.323 | 23.220 | 39.835 | 0.903±0.069 | 0.983±0.069 | ＜0.001 | 0.819 |  | 0.748 |  |
| 4 | 21.394±2.630 | 15.488 | 28.417 | 0.894±0.081 | 0.980±0.081 | ＜0.001 | 0.796 |  | 0.720 |  |
| 5 | 14.583±1.868 | 10.041 | 19.397 | 0.858±0.098 | 0.989±0.098 | ＜0.001 | 0.790 |  | 0.698 |  |
| 6 | 62.049±3.572 | 49.426 | 75.904 | 0.911±0.057 | 0.985±0.057 | ＜0.001 | 0.846 |  | 0.790 |  |
| 7 | 28.058±2.901 | 20.695 | 36.802 | 0.885±0.080 | 0.987±0.080 | ＜0.001 | 0.812 |  | 0.727 |  |
| 8 | 33.723±5.552 | 23.157 | 44.572 | 0.875±0.123 | 0.988±0.123 | ＜0.001 | 0.797 |  | 0.706 |  |
| 9 | 7.189±4.462 | 1.187 | 13.539 | 0.685±0.343 | 0.972±0.343 | ＜0.001 | 0.464 |  | 0.366 |  |
| 10 | 29.859±2.882 | 21.143 | 39.549 | 0.877±0.070 | 0.986±0.070 | ＜0.001 | 0.799 |  | 0.712 |  |
| 11 | 27.974±1.327 | 21.991 | 35.166 | 0.917±0.056 | 0.989±0.056 | ＜0.001 | 0.850 |  | 0.775 |  |
| 12 | 43.771±17.350 | 24.279 | 82.481 | 0.885±0.084 | 0.985±0.084 | ＜0.001 | 0.639 |  | 0.490 |  |
| 13 | 31.043±3.530 | 22.895 | 40.515 | 0.887±0.078 | 0.987±0.078 | ＜0.001 | 0.814 |  | 0.730 |  |
| 14 | 26.319±3.810 | 19.659 | 34.605 | 0.898±0.073 | 0.984±0.073 | ＜0.001 | 0.813 |  | 0.733 |  |
| 15 | 21.295±2.729 | 15.071 | 28.157 | 0.885±0.079 | 0.987±0.079 | ＜0.001 | 0.804 |  | 0.716 |  |
| 16 | 42.713±4.207 | 33.292 | 52.245 | 0.912±0.072 | 0.984±0.072 | ＜0.001 | 0.841 |  | 0.785 |  |
| 17 | 33.580±1.891 | 26.088 | 42.884 | 0.917±0.051 | 0.989±0.051 | ＜0.001 | 0.844 |  | 0.764 |  |
| 18 | 16.936±3.343 | 10.235 | 23.276 | 0.849±0.154 | 0.985±0.154 | ＜0.001 | 0.753 |  | 0.657 |  |
| 19 | 38.934±2.224 | 31.313 | 47.974 | 0.923±0.039 | 0.986±0.039 | ＜0.001 | 0.855 |  | 0.792 |  |
| 20 | 13.366±3.237 | 8.038 | 18.362 | 0.829±0.165 | 0.988±0.165 | ＜0.001 | 0.748 |  | 0.649 |  |
| 21 | 42.252±4.321 | 31.659 | 54.958 | 0.900±0.077 | 0.986±0.077 | ＜0.001 | 0.821 |  | 0.740 |  |
| 22 | 32.149±3.987 | 24.081 | 41.075 | 0.898±0.069 | 0.987±0.069 | ＜0.001 | 0.826 |  | 0.749 |  |
| 23 | 14.286±2.563 | 8.571 | 20.093 | 0.827±0.113 | 0.986±0.113 | ＜0.001 | 0.737 |  | 0.637 |  |
| 24 | 25.448±4.883 | 17.260 | 34.234 | 0.875±0.125 | 0.983±0.125 | ＜0.001 | 0.780 |  | 0.695 |  |
| 25 | 23.656±3.186 | 16.745 | 31.064 | 0.879±0.089 | 0.991±0.089 | ＜0.001 | 0.812 |  | 0.717 |  |
| 26 | 16.659±2.441 | 11.393 | 22.781 | 0.882±0.098 | 0.984±0.098 | ＜0.001 | 0.784 |  | 0.691 |  |
| 27 | 14.883±2.030 | 10.464 | 19.622 | 0.878±0.108 | 0.981±0.108 | ＜0.001 | 0.786 |  | 0.712 |  |
| 28 | 11.884±2.940 | 7.299 | 17.533 | 0.832±0.109 | 0.979±0.109 | ＜0.001 | 0.717 |  | 0.617 |  |
| 29 | 37.488±3.242 | 28.216 | 46.565 | 0.902±0.079 | 0.987±0.079 | ＜0.001 | 0.834 |  | 0.766 |  |
| 30 | 30.691±2.650 | 23.439 | 38.969 | 0.908±0.069 | 0.984±0.069 | ＜0.001 | 0.828 |  | 0.759 |  |

| Case | volume | Intersection volume | Union volume | Sensitivity | Specificity | p-value: | Overall kappa: | Mean±SD | CI-gen | Mean±SD |
| --- | --- | --- | --- | --- | --- | --- | --- | --- | --- | --- |
| 1 | 3.100±1.243 | 1.302 | 4.754 | 0.767±0.271 | 0.978±0.271 | ＜0.001 | 0.624 | 0.670±0.094 | 0.532 | 0.602±0.100 |
| 2 | 3.822±1.306 | 2.103 | 5.690 | 0.808±0.191 | 0.958±0.191 | ＜0.001 | 0.635 |  | 0.588 |  |
| 3 | 8.517±1.626 | 5.036 | 11.686 | 0.851±0.118 | 0.963±0.118 | ＜0.001 | 0.697 |  | 0.654 |  |
| 4 | 1.893±1.007 | 0.164 | 3.835 | 0.631±0.317 | 0.939±0.317 | ＜0.001 | 0.331 |  | 0.303 |  |
| 5 | 5.001±2.210 | 2.165 | 9.413 | 0.756±0.172 | 0.960±0.172 | ＜0.001 | 0.536 |  | 0.433 |  |
| 6 | 8.800±3.938 | 3.384 | 14.847 | 0.750±0.230 | 0.957±0.230 | ＜0.001 | 0.542 |  | 0.474 |  |
| 7 | 6.842±1.739 | 4.020 | 9.318 | 0.827±0.183 | 0.979±0.183 | ＜0.001 | 0.715 |  | 0.649 |  |
| 8 | 7.869±1.473 | 5.276 | 10.467 | 0.862±0.140 | 0.978±0.140 | ＜0.001 | 0.760 |  | 0.693 |  |
| 9 | 5.792±2.274 | 2.317 | 9.465 | 0.758±0.244 | 0.971±0.244 | ＜0.001 | 0.589 |  | 0.495 |  |
| 10 | 8.494±1.390 | 5.621 | 11.817 | 0.870±0.097 | 0.970±0.097 | ＜0.001 | 0.742 |  | 0.669 |  |
| 11 | 7.006±1.375 | 4.564 | 9.455 | 0.869±0.128 | 0.949±0.128 | ＜0.001 | 0.702 |  | 0.680 |  |
| 12 | 7.646±1.589 | 4.992 | 10.388 | 0.858±0.143 | 0.959±0.143 | ＜0.001 | 0.711 |  | 0.673 |  |
| 13 | 8.760±2.354 | 3.652 | 12.790 | 0.779±0.174 | 0.980±0.174 | ＜0.001 | 0.640 |  | 0.560 |  |
| 14 | 7.647±1.068 | 5.220 | 10.067 | 0.874±0.094 | 0.979±0.094 | ＜0.001 | 0.775 |  | 0.707 |  |
| 15 | 7.846±4.142 | 4.117 | 15.187 | 0.871±0.133 | 0.974±0.133 | ＜0.001 | 0.601 |  | 0.465 |  |
| 16 | 9.670±1.682 | 4.994 | 13.817 | 0.792±0.119 | 0.976±0.119 | ＜0.001 | 0.662 |  | 0.597 |  |
| 17 | 6.100±1.151 | 4.048 | 8.192 | 0.856±0.138 | 0.994±0.138 | ＜0.001 | 0.796 |  | 0.687 |  |
| 18 | 5.868±0.719 | 4.135 | 7.790 | 0.868±0.073 | 0.972±0.073 | ＜0.001 | 0.761 |  | 0.704 |  |
| 19 | 9.875±4.937 | 4.873 | 19.411 | 0.875±0.134 | 0.967±0.134 | ＜0.001 | 0.580 |  | 0.452 |  |
| 20 | 4.188±1.127 | 2.006 | 6.101 | 0.809±0.184 | 0.967±0.184 | ＜0.001 | 0.643 |  | 0.584 |  |
| 21 | 5.758±1.501 | 2.976 | 8.369 | 0.827±0.176 | 0.969±0.176 | ＜0.001 | 0.671 |  | 0.600 |  |
| 22 | 6.829±1.314 | 4.395 | 9.302 | 0.849±0.139 | 0.966±0.139 | ＜0.001 | 0.716 |  | 0.668 |  |
| 23 | 7.556±1.600 | 4.875 | 9.956 | 0.851±0.160 | 0.981±0.160 | ＜0.001 | 0.754 |  | 0.688 |  |
| 24 | 6.593±1.573 | 4.150 | 9.338 | 0.844±0.154 | 0.962±0.154 | ＜0.001 | 0.700 |  | 0.640 |  |
| 25 | 8.602±1.937 | 5.355 | 11.605 | 0.855±0.160 | 0.972±0.160 | ＜0.001 | 0.729 |  | 0.671 |  |
| 26 | 4.758±1.308 | 2.498 | 6.858 | 0.803±0.186 | 0.964±0.186 | ＜0.001 | 0.642 |  | 0.596 |  |
| 27 | 3.235±0.539 | 2.028 | 4.594 | 0.853±0.110 | 0.966±0.110 | ＜0.001 | 0.712 |  | 0.643 |  |
| 28 | 2.524±0.903 | 1.340 | 3.985 | 0.834±0.196 | 0.952±0.196 | ＜0.001 | 0.632 |  | 0.562 |  |
| 29 | 9.815±1.519 | 6.469 | 12.722 | 0.872±0.110 | 0.977±0.110 | ＜0.001 | 0.765 |  | 0.708 |  |
| 30 | 7.389±1.632 | 4.717 | 9.921 | 0.844±0.159 | 0.972±0.159 | ＜0.001 | 0.723 |  | 0.673 |  |

TableS14：Submandibular Gland L

The mean volumes (± SD) of the auto-contours of the Submandibular Gland-L for each patient, as well as the intersection and union volumes, sensitivity, specificity, the overall Kappa coefficient with its p-value (H₀: κ = 0), and the generalized conformity index (CI-gen) for each case. The overall Kappa coefficient with its p-value (H₀: κ = 0); p < 0.001 indicates agreement significantly higher than random.

TableS15：Submandibular Gland R

| Case | volume | Intersection volume | Union volume | Sensitivity | Specificity | p-value: | Overall kappa: | Mean±SD | CI-gen | Mean±SD |
| --- | --- | --- | --- | --- | --- | --- | --- | --- | --- | --- |
| 1 | 1.813±0.290 | 0.288 | 4.037 | 0.681±0.281 | 0.958±0.281 | ＜0.001 | 0.381 | 0.680±0.088 | 0.294 | 0.617±0.098 |
| 2 | 4.445±0.898 | 2.765 | 5.964 | 0.859±0.144 | 0.972±0.144 | ＜0.001 | 0.733 |  | 0.676 |  |
| 3 | 8.262±1.503 | 5.101 | 10.863 | 0.850±0.138 | 0.968±0.138 | ＜0.001 | 0.712 |  | 0.681 |  |
| 4 | 1.687±0.630 | 0.839 | 2.780 | 0.803±0.138 | 0.937±0.138 | ＜0.001 | 0.570 |  | 0.525 |  |
| 5 | 4.654±1.797 | 2.432 | 7.269 | 0.799±0.191 | 0.971±0.191 | ＜0.001 | 0.647 |  | 0.558 |  |
| 6 | 10.352±1.922 | 5.596 | 14.481 | 0.833±0.136 | 0.969±0.136 | ＜0.001 | 0.686 |  | 0.626 |  |
| 7 | 6.155±1.417 | 3.212 | 9.401 | 0.816±0.148 | 0.960±0.148 | ＜0.001 | 0.641 |  | 0.572 |  |
| 8 | 8.909±1.533 | 6.234 | 11.436 | 0.885±0.125 | 0.978±0.125 | ＜0.001 | 0.785 |  | 0.728 |  |
| 9 | 3.856±1.287 | 1.664 | 6.490 | 0.770±0.160 | 0.964±0.160 | ＜0.001 | 0.584 |  | 0.492 |  |
| 10 | 9.084±2.353 | 5.025 | 13.802 | 0.853±0.169 | 0.947±0.169 | ＜0.001 | 0.648 |  | 0.589 |  |
| 11 | 6.491±1.490 | 3.889 | 9.075 | 0.824±0.168 | 0.980±0.168 | ＜0.001 | 0.718 |  | 0.637 |  |
| 12 | 6.920±3.020 | 3.488 | 11.654 | 0.769±0.228 | 0.972±0.228 | ＜0.001 | 0.610 |  | 0.503 |  |
| 13 | 10.325±1.747 | 5.870 | 14.198 | 0.833±0.129 | 0.978±0.129 | ＜0.001 | 0.715 |  | 0.642 |  |
| 14 | 6.457±1.357 | 3.447 | 9.065 | 0.817±0.146 | 0.976±0.146 | ＜0.001 | 0.687 |  | 0.618 |  |
| 15 | 5.838±1.241 | 3.736 | 7.700 | 0.867±0.159 | 0.969±0.159 | ＜0.001 | 0.736 |  | 0.692 |  |
| 16 | 9.693±1.683 | 5.795 | 13.231 | 0.844±0.115 | 0.973±0.115 | ＜0.001 | 0.718 |  | 0.656 |  |
| 17 | 7.018±1.403 | 4.376 | 9.573 | 0.851±0.140 | 0.964±0.140 | ＜0.001 | 0.708 |  | 0.664 |  |
| 18 | 5.518±0.930 | 4.495 | 6.540 | 0.903±0.083 | 0.955±0.083 | ＜0.001 | 0.728 |  | 0.671 |  |
| 19 | 8.187±1.740 | 5.442 | 10.605 | 0.880±0.158 | 0.968±0.158 | ＜0.001 | 0.749 |  | 0.711 |  |
| 20 | 4.820±1.565 | 2.101 | 8.409 | 0.763±0.166 | 0.949±0.166 | ＜0.001 | 0.544 |  | 0.471 |  |
| 21 | 8.071±1.976 | 4.548 | 11.279 | 0.826±0.177 | 0.972±0.177 | ＜0.001 | 0.692 |  | 0.629 |  |
| 22 | 7.869±1.573 | 5.007 | 10.389 | 0.860±0.152 | 0.973±0.152 | ＜0.001 | 0.737 |  | 0.688 |  |
| 23 | 6.577±1.681 | 3.794 | 8.669 | 0.843±0.196 | 0.980±0.196 | ＜0.001 | 0.727 |  | 0.669 |  |
| 24 | 7.427±1.501 | 4.740 | 9.872 | 0.849±0.149 | 0.977±0.149 | ＜0.001 | 0.740 |  | 0.681 |  |
| 25 | 10.286±2.241 | 6.793 | 14.382 | 0.868±0.087 | 0.975±0.087 | ＜0.001 | 0.747 |  | 0.669 |  |
| 26 | 6.719±1.255 | 4.328 | 9.232 | 0.848±0.119 | 0.974±0.119 | ＜0.001 | 0.733 |  | 0.665 |  |
| 27 | 4.123±0.657 | 2.704 | 5.603 | 0.852±0.110 | 0.974±0.110 | ＜0.001 | 0.740 |  | 0.675 |  |
| 28 | 2.478±1.312 | 0.929 | 4.584 | 0.759±0.253 | 0.948±0.253 | ＜0.001 | 0.508 |  | 0.430 |  |
| 29 | 9.429±1.323 | 6.696 | 12.374 | 0.904±0.066 | 0.945±0.066 | ＜0.001 | 0.740 |  | 0.721 |  |
| 30 | 6.906±1.553 | 4.354 | 9.491 | 0.849±0.144 | 0.974±0.144 | ＜0.001 | 0.731 |  | 0.662 |  |

The mean volumes (± SD) of the auto-contours of the Submandibular Gland-R for each patient, as well as the intersection and union volumes, sensitivity, specificity, the overall Kappa coefficient with its p-value (H₀: κ = 0), and the generalized conformity index (CI-gen) for each case. The overall Kappa coefficient with its p-value (H₀: κ = 0); p < 0.001 indicates agreement significantly higher than random.

| Case | volume | Intersection volume | Union volume | Sensitivity | Specificity | p-value: | Overall kappa: | Mean±SD | CI-gen | Mean±SD |
| --- | --- | --- | --- | --- | --- | --- | --- | --- | --- | --- |
| 1 | 78.406±9.080 | 57.669 | 103.595 | 0.891±0.059 | 0.995±0.004 | ＜0.001 | 0.831 | 0.784±0.018 | 0.726 | 0.667±0.022 |
| 2 | 21.988±4.185 | 14.118 | 30.129 | 0.861±0.127 | 0.992±0.008 | ＜0.001 | 0.784 |  | 0.673 |  |
| 3 | 23.643±4.170 | 15.867 | 33.397 | 0.857±0.090 | 0.994±0.006 | ＜0.001 | 0.785 |  | 0.663 |  |
| 4 | 27.270±5.488 | 17.628 | 37.230 | 0.867±0.133 | 0.993±0.007 | ＜0.001 | 0.790 |  | 0.678 |  |
| 5 | 23.704±3.158 | 15.632 | 33.820 | 0.870±0.079 | 0.991±0.008 | ＜0.001 | 0.775 |  | 0.659 |  |
| 6 | 27.952±5.367 | 18.829 | 39.237 | 0.868±0.110 | 0.994±0.006 | ＜0.001 | 0.789 |  | 0.670 |  |
| 7 | 20.032±3.173 | 11.843 | 30.237 | 0.823±0.070 | 0.995±0.004 | ＜0.001 | 0.739 |  | 0.600 |  |
| 8 | 23.012±4.070 | 14.545 | 32.603 | 0.843±0.097 | 0.994±0.006 | ＜0.001 | 0.771 |  | 0.647 |  |
| 9 | 25.690±4.402 | 16.602 | 36.632 | 0.861±0.101 | 0.994±0.006 | ＜0.001 | 0.776 |  | 0.654 |  |
| 10 | 31.006±6.741 | 20.377 | 43.698 | 0.848±0.123 | 0.995±0.006 | ＜0.001 | 0.781 |  | 0.657 |  |
| 11 | 33.855±6.012 | 22.408 | 45.841 | 0.855±0.126 | 0.996±0.003 | ＜0.001 | 0.799 |  | 0.682 |  |
| 12 | 24.771±3.934 | 16.703 | 35.541 | 0.871±0.080 | 0.991±0.008 | ＜0.001 | 0.777 |  | 0.660 |  |
| 13 | 27.331±4.669 | 18.445 | 39.582 | 0.866±0.068 | 0.996±0.004 | ＜0.001 | 0.784 |  | 0.655 |  |
| 14 | 24.357±4.326 | 15.373 | 34.354 | 0.847±0.117 | 0.993±0.006 | ＜0.001 | 0.772 |  | 0.651 |  |
| 15 | 25.841±3.662 | 17.812 | 36.236 | 0.881±0.072 | 0.994±0.006 | ＜0.001 | 0.797 |  | 0.680 |  |
| 16 | 27.641±5.184 | 18.796 | 40.016 | 0.886±0.096 | 0.991±0.009 | ＜0.001 | 0.780 |  | 0.661 |  |
| 17 | 23.835±4.582 | 15.606 | 33.861 | 0.860±0.090 | 0.993±0.007 | ＜0.001 | 0.778 |  | 0.657 |  |
| 18 | 30.969±5.403 | 21.096 | 42.052 | 0.869±0.114 | 0.993±0.007 | ＜0.001 | 0.799 |  | 0.690 |  |
| 19 | 24.773±4.144 | 16.537 | 35.365 | 0.863±0.079 | 0.992±0.008 | ＜0.001 | 0.778 |  | 0.659 |  |
| 20 | 18.906±2.135 | 12.510 | 26.845 | 0.864±0.054 | 0.995±0.004 | ＜0.001 | 0.785 |  | 0.661 |  |
| 21 | 29.799±4.859 | 20.692 | 40.420 | 0.876±0.108 | 0.996±0.004 | ＜0.001 | 0.812 |  | 0.697 |  |
| 22 | 33.882±2.594 | 22.440 | 45.516 | 0.856±0.053 | 0.993±0.004 | ＜0.001 | 0.795 |  | 0.687 |  |
| 23 | 26.638±4.519 | 17.654 | 38.242 | 0.869±0.075 | 0.990±0.010 | ＜0.001 | 0.773 |  | 0.657 |  |
| 24 | 19.664±3.376 | 12.996 | 27.744 | 0.865±0.091 | 0.991±0.009 | ＜0.001 | 0.779 |  | 0.664 |  |
| 25 | 32.666±6.217 | 21.679 | 45.262 | 0.869±0.126 | 0.996±0.004 | ＜0.001 | 0.796 |  | 0.676 |  |
| 26 | 22.548±3.621 | 14.356 | 32.178 | 0.860±0.089 | 0.991±0.009 | ＜0.001 | 0.769 |  | 0.651 |  |
| 27 | 24.718±3.686 | 16.633 | 34.278 | 0.868±0.073 | 0.994±0.006 | ＜0.001 | 0.795 |  | 0.677 |  |
| 28 | 24.934±5.349 | 16.755 | 33.313 | 0.879±0.130 | 0.982±0.015 | ＜0.001 | 0.742 |  | 0.638 |  |
| 29 | 25.296±5.018 | 16.972 | 33.847 | 0.862±0.137 | 0.995±0.005 | ＜0.001 | 0.804 |  | 0.692 |  |
| 30 | 28.557±6.174 | 18.708 | 39.219 | 0.868±0.150 | 0.993±0.007 | ＜0.001 | 0.792 |  | 0.677 |  |

TableS16：Spinal Cord

The mean volumes (± SD) of the auto-contours of the Spinal Cord for each patient, as well as the intersection and union volumes, sensitivity, specificity, the overall Kappa coefficient with its p-value (H₀: κ = 0), and the generalized conformity index (CI-gen) for each case. The overall Kappa coefficient with its p-value (H₀: κ = 0); p < 0.001 indicates agreement significantly higher than random.

| Case | volume | Intersection volume | Union volume | Sensitivity | Specificity | p-value: | Overall kappa: | Mean±SD | CI-gen | Mean±SD |
| --- | --- | --- | --- | --- | --- | --- | --- | --- | --- | --- |
| 1 | 83.021±16.380 | 49.363 | 113.369 | 0.832±0.167 | 0.979±0.167 | ＜0.001 | 0.720 | 0.689±0.046 | 0.650 | 0.612±0.056 |
| 2 | 59.023±18.441 | 26.755 | 88.233 | 0.792±0.244 | 0.974±0.244 | ＜0.001 | 0.641 |  | 0.560 |  |
| 3 | 99.478±19.682 | 60.805 | 131.288 | 0.827±0.165 | 0.979±0.165 | ＜0.001 | 0.712 |  | 0.669 |  |
| 4 | 65.003±20.750 | 30.496 | 94.997 | 0.786±0.231 | 0.993±0.231 | ＜0.001 | 0.703 |  | 0.573 |  |
| 5 | 87.140±9.294 | 40.734 | 145.610 | 0.812±0.219 | 0.979±0.219 | ＜0.001 | 0.640 |  | 0.517 |  |
| 6 | 90.383±17.629 | 55.419 | 119.409 | 0.841±0.174 | 0.978±0.174 | ＜0.001 | 0.727 |  | 0.674 |  |
| 7 | 100.427±24.319 | 58.811 | 137.181 | 0.839±0.182 | 0.974±0.182 | ＜0.001 | 0.711 |  | 0.650 |  |
| 8 | 71.963±15.949 | 38.979 | 101.792 | 0.806±0.190 | 0.993±0.190 | ＜0.001 | 0.735 |  | 0.613 |  |
| 9 | 81.239±24.234 | 39.731 | 116.128 | 0.795±0.214 | 0.977±0.214 | ＜0.001 | 0.660 |  | 0.590 |  |
| 10 | 87.927±17.877 | 50.834 | 118.100 | 0.839±0.171 | 0.981±0.171 | ＜0.001 | 0.729 |  | 0.657 |  |
| 11 | 68.996±20.345 | 32.839 | 98.654 | 0.796±0.219 | 0.974±0.219 | ＜0.001 | 0.651 |  | 0.587 |  |
| 12 | 90.230±13.759 | 59.209 | 118.910 | 0.855±0.123 | 0.971±0.123 | ＜0.001 | 0.734 |  | 0.691 |  |
| 13 | 79.869±16.457 | 50.384 | 106.905 | 0.827±0.162 | 0.981±0.162 | ＜0.001 | 0.728 |  | 0.666 |  |
| 14 | 82.742±16.434 | 48.646 | 110.920 | 0.824±0.173 | 0.994±0.173 | ＜0.001 | 0.766 |  | 0.656 |  |
| 15 | 98.034±15.122 | 52.277 | 146.290 | 0.817±0.193 | 0.987±0.193 | ＜0.001 | 0.707 |  | 0.588 |  |
| 16 | 78.011±12.309 | 49.468 | 103.631 | 0.853±0.162 | 0.976±0.162 | ＜0.001 | 0.739 |  | 0.681 |  |
| 17 | 98.012±20.618 | 55.542 | 133.918 | 0.834±0.181 | 0.976±0.181 | ＜0.001 | 0.706 |  | 0.644 |  |
| 18 | 57.492±23.719 | 19.791 | 89.458 | 0.730±0.284 | 0.978±0.284 | ＜0.001 | 0.571 |  | 0.495 |  |
| 19 | 102.890±14.048 | 58.124 | 168.243 | 0.842±0.160 | 0.976±0.160 | ＜0.001 | 0.673 |  | 0.557 |  |
| 20 | 88.272±21.572 | 49.324 | 123.234 | 0.823±0.195 | 0.976±0.195 | ＜0.001 | 0.697 |  | 0.628 |  |
| 21 | 91.489±19.154 | 55.911 | 124.668 | 0.845±0.170 | 0.972±0.170 | ＜0.001 | 0.718 |  | 0.659 |  |
| 22 | 86.877±24.514 | 41.650 | 123.764 | 0.802±0.224 | 0.976±0.224 | ＜0.001 | 0.659 |  | 0.592 |  |
| 23 | 83.648±20.065 | 44.181 | 120.320 | 0.813±0.189 | 0.974±0.189 | ＜0.001 | 0.678 |  | 0.602 |  |
| 24 | 82.384±15.617 | 52.441 | 111.053 | 0.835±0.155 | 0.975±0.155 | ＜0.001 | 0.722 |  | 0.667 |  |
| 25 | 82.238±18.640 | 42.685 | 113.327 | 0.823±0.202 | 0.979±0.202 | ＜0.001 | 0.696 |  | 0.625 |  |
| 26 | 81.418±18.883 | 44.800 | 115.345 | 0.834±0.182 | 0.969±0.182 | ＜0.001 | 0.685 |  | 0.622 |  |
| 27 | 63.406±20.065 | 30.383 | 90.674 | 0.793±0.237 | 0.977±0.237 | ＜0.001 | 0.658 |  | 0.587 |  |
| 28 | 59.063±22.721 | 23.949 | 89.273 | 0.749±0.264 | 0.978±0.264 | ＜0.001 | 0.603 |  | 0.528 |  |
| 29 | 110.126±8.446 | 49.324 | 195.634 | 0.808±0.214 | 0.972±0.214 | ＜0.001 | 0.600 |  | 0.482 |  |
| 30 | 84.508±19.372 | 46.941 | 115.330 | 0.832±0.189 | 0.974±0.189 | ＜0.001 | 0.698 |  | 0.641 |  |

TableS17：Temporal Lobe L

The mean volumes (± SD) of the auto-contours of the Temporal Lobe-L for each patient, as well as the intersection and union volumes, sensitivity, specificity, the overall Kappa coefficient with its p-value (H₀: κ = 0), and the generalized conformity index (CI-gen) for each case. The overall Kappa coefficient with its p-value (H₀: κ = 0); p < 0.001 indicates agreement significantly higher than random.

| Case | volume | Intersection volume | Union volume | Sensitivity | Specificity | p-value: | Overall kappa: | Mean±SD | CI-gen | Mean±SD |
| --- | --- | --- | --- | --- | --- | --- | --- | --- | --- | --- |
| 1 | 74.648±17.489 | 38.480 | 107.951 | 0.828±0.184 | 0.973±0.184 | ＜0.001 | 0.683 | 0.692±0.044 | 0.602 | 0.626±0.052 |
| 2 | 74.023±19.538 | 38.925 | 106.222 | 0.813±0.216 | 0.975±0.216 | ＜0.001 | 0.678 |  | 0.603 |  |
| 3 | 95.481±17.826 | 52.470 | 130.450 | 0.854±0.148 | 0.959±0.148 | ＜0.001 | 0.682 |  | 0.646 |  |
| 4 | 70.998±19.803 | 36.337 | 102.140 | 0.808±0.208 | 0.977±0.208 | ＜0.001 | 0.678 |  | 0.597 |  |
| 5 | 71.933±21.569 | 29.448 | 109.128 | 0.799±0.262 | 0.972±0.262 | ＜0.001 | 0.630 |  | 0.545 |  |
| 6 | 81.718±20.343 | 47.135 | 109.060 | 0.827±0.191 | 0.979±0.191 | ＜0.001 | 0.709 |  | 0.655 |  |
| 7 | 111.489±27.935 | 65.590 | 152.964 | 0.846±0.189 | 0.973±0.189 | ＜0.001 | 0.713 |  | 0.652 |  |
| 8 | 94.706±23.873 | 55.929 | 128.600 | 0.821±0.196 | 0.979±0.196 | ＜0.001 | 0.709 |  | 0.647 |  |
| 9 | 86.122±24.623 | 40.855 | 126.500 | 0.796±0.221 | 0.975±0.221 | ＜0.001 | 0.653 |  | 0.575 |  |
| 10 | 89.188±16.820 | 54.730 | 119.212 | 0.860±0.175 | 0.978±0.175 | ＜0.001 | 0.747 |  | 0.676 |  |
| 11 | 84.820±19.844 | 50.214 | 115.733 | 0.841±0.186 | 0.977±0.186 | ＜0.001 | 0.722 |  | 0.654 |  |
| 12 | 93.490±8.166 | 59.819 | 122.832 | 0.858±0.114 | 0.971±0.114 | ＜0.001 | 0.732 |  | 0.690 |  |
| 13 | 82.258±9.980 | 57.442 | 111.551 | 0.893±0.092 | 0.962±0.092 | ＜0.001 | 0.754 |  | 0.698 |  |
| 14 | 87.158±16.817 | 55.065 | 117.892 | 0.866±0.154 | 0.971±0.154 | ＜0.001 | 0.736 |  | 0.676 |  |
| 15 | 77.200±15.836 | 39.762 | 107.883 | 0.829±0.189 | 0.974±0.189 | ＜0.001 | 0.687 |  | 0.619 |  |
| 16 | 82.659±15.523 | 54.953 | 109.389 | 0.863±0.152 | 0.974±0.152 | ＜0.001 | 0.749 |  | 0.694 |  |
| 17 | 93.848±17.365 | 56.225 | 130.288 | 0.867±0.153 | 0.971±0.153 | ＜0.001 | 0.726 |  | 0.656 |  |
| 18 | 67.789±26.173 | 27.376 | 105.364 | 0.776±0.264 | 0.971±0.264 | ＜0.001 | 0.606 |  | 0.525 |  |
| 19 | 86.901±17.252 | 53.390 | 116.248 | 0.843±0.159 | 0.978±0.159 | ＜0.001 | 0.731 |  | 0.670 |  |
| 20 | 85.701±20.479 | 44.027 | 124.086 | 0.845±0.193 | 0.968±0.193 | ＜0.001 | 0.679 |  | 0.607 |  |
| 21 | 102.258±21.678 | 63.184 | 139.962 | 0.869±0.165 | 0.969±0.165 | ＜0.001 | 0.728 |  | 0.668 |  |
| 22 | 87.952±25.868 | 40.727 | 131.346 | 0.806±0.239 | 0.972±0.239 | ＜0.001 | 0.649 |  | 0.567 |  |
| 23 | 81.301±22.957 | 41.586 | 120.706 | 0.843±0.195 | 0.961±0.195 | ＜0.001 | 0.662 |  | 0.592 |  |
| 24 | 89.032±23.349 | 49.778 | 126.927 | 0.844±0.188 | 0.964±0.188 | ＜0.001 | 0.685 |  | 0.623 |  |
| 25 | 99.989±21.199 | 58.128 | 135.945 | 0.840±0.184 | 0.982±0.184 | ＜0.001 | 0.732 |  | 0.653 |  |
| 26 | 85.554±18.423 | 47.231 | 120.612 | 0.852±0.182 | 0.968±0.182 | ＜0.001 | 0.696 |  | 0.631 |  |
| 27 | 69.584±26.240 | 29.369 | 102.454 | 0.790±0.281 | 0.973±0.281 | ＜0.001 | 0.629 |  | 0.559 |  |
| 28 | 61.053±26.002 | 21.162 | 97.035 | 0.748±0.285 | 0.972±0.285 | ＜0.001 | 0.574 |  | 0.494 |  |
| 29 | 96.813±24.044 | 53.769 | 138.761 | 0.857±0.165 | 0.960±0.165 | ＜0.001 | 0.683 |  | 0.625 |  |
| 30 | 82.404±17.149 | 50.875 | 111.416 | 0.874±0.156 | 0.968±0.156 | ＜0.001 | 0.730 |  | 0.676 |  |

TableS18：Temporal Lobe R

The mean volumes (± SD) of the auto-contours of the Temporal Lobe-R for each patient, as well as the intersection and union volumes, sensitivity, specificity, the overall Kappa coefficient with its p-value (H₀: κ = 0), and the generalized conformity index (CI-gen) for each case. The overall Kappa coefficient with its p-value (H₀: κ = 0); p < 0.001 indicates agreement significantly higher than random.

| Case | volume | Intersection volume | Union volume | Sensitivity | Specificity | p-value: | Overall kappa: | Mean±SD | CI-gen | Mean±SD |
| --- | --- | --- | --- | --- | --- | --- | --- | --- | --- | --- |
| 1 | 1.595±1.309 | 0.000 | 3.839 | 0.566±0.426 | 0.898±0.426 | ＜0.001 | 0.171 | 0.434±0.101 | 0.194 | 0.380±0.088 |
| 2 | 1.548±0.866 | 0.315 | 3.018 | 0.695±0.368 | 0.937±0.368 | ＜0.001 | 0.385 |  | 0.355 |  |
| 3 | 3.130±0.453 | 1.367 | 5.611 | 0.845±0.188 | 0.909±0.188 | ＜0.001 | 0.514 |  | 0.461 |  |
| 4 | 1.549±0.769 | 0.288 | 3.256 | 0.662±0.329 | 0.948±0.329 | ＜0.001 | 0.373 |  | 0.314 |  |
| 5 | 2.292±0.852 | 0.408 | 5.418 | 0.622±0.252 | 0.985±0.252 | ＜0.001 | 0.386 |  | 0.260 |  |
| 6 | 1.922±0.929 | 0.633 | 4.325 | 0.684±0.241 | 0.939±0.241 | ＜0.001 | 0.381 |  | 0.317 |  |
| 7 | 2.671±0.486 | 1.225 | 4.886 | 0.775±0.160 | 0.942±0.160 | ＜0.001 | 0.528 |  | 0.458 |  |
| 8 | 2.346±0.597 | 0.857 | 4.784 | 0.773±0.215 | 0.908±0.215 | ＜0.001 | 0.420 |  | 0.372 |  |
| 9 | 2.012±0.480 | 0.581 | 4.085 | 0.715±0.278 | 0.934±0.278 | ＜0.001 | 0.418 |  | 0.350 |  |
| 10 | 3.505±1.392 | 0.703 | 7.319 | 0.637±0.253 | 0.944±0.253 | ＜0.001 | 0.356 |  | 0.313 |  |
| 11 | 2.233±0.393 | 0.908 | 4.115 | 0.765±0.233 | 0.942±0.233 | ＜0.001 | 0.512 |  | 0.432 |  |
| 12 | 1.496±0.216 | 0.305 | 3.204 | 0.721±0.198 | 0.917±0.198 | ＜0.001 | 0.351 |  | 0.320 |  |
| 13 | 2.514±0.653 | 1.060 | 4.487 | 0.777±0.214 | 0.914±0.214 | ＜0.001 | 0.491 |  | 0.447 |  |
| 14 | 2.541±0.481 | 1.230 | 4.589 | 0.826±0.210 | 0.933±0.210 | ＜0.001 | 0.545 |  | 0.485 |  |
| 15 | 1.823±0.703 | 0.714 | 3.909 | 0.804±0.201 | 0.943±0.201 | ＜0.001 | 0.464 |  | 0.373 |  |
| 16 | 2.980±0.694 | 1.443 | 5.313 | 0.760±0.139 | 0.931±0.139 | ＜0.001 | 0.516 |  | 0.466 |  |
| 17 | 3.612±1.341 | 1.238 | 8.493 | 0.793±0.216 | 0.981±0.216 | ＜0.001 | 0.456 |  | 0.316 |  |
| 18 | 2.164±0.562 | 0.810 | 4.300 | 0.775±0.170 | 0.912±0.170 | ＜0.001 | 0.440 |  | 0.391 |  |
| 19 | 1.682±0.828 | 0.357 | 3.643 | 0.648±0.236 | 0.925±0.236 | ＜0.001 | 0.327 |  | 0.309 |  |
| 20 | 2.084±0.611 | 0.847 | 4.149 | 0.752±0.258 | 0.935±0.258 | ＜0.001 | 0.462 |  | 0.401 |  |
| 21 | 1.768±0.564 | 0.681 | 3.432 | 0.747±0.202 | 0.926±0.202 | ＜0.001 | 0.458 |  | 0.402 |  |
| 22 | 1.834±0.382 | 0.459 | 3.662 | 0.705±0.197 | 0.940±0.197 | ＜0.001 | 0.413 |  | 0.361 |  |
| 23 | 1.881±0.445 | 0.749 | 3.612 | 0.788±0.207 | 0.908±0.207 | ＜0.001 | 0.449 |  | 0.428 |  |
| 24 | 1.830±0.273 | 0.621 | 3.330 | 0.740±0.173 | 0.945±0.173 | ＜0.001 | 0.490 |  | 0.431 |  |
| 25 | 2.395±0.879 | 0.753 | 4.199 | 0.723±0.261 | 0.925±0.261 | ＜0.001 | 0.436 |  | 0.430 |  |
| 26 | 2.125±0.829 | 0.803 | 4.504 | 0.811±0.174 | 0.874±0.174 | ＜0.001 | 0.373 |  | 0.357 |  |
| 27 | 1.245±0.635 | 0.205 | 2.761 | 0.674±0.378 | 0.939±0.378 | ＜0.001 | 0.332 |  | 0.287 |  |
| 28 | 2.227±0.943 | 0.300 | 4.832 | 0.597±0.220 | 0.952±0.220 | ＜0.001 | 0.329 |  | 0.277 |  |
| 29 | 13.820±3.249 | 8.752 | 19.336 | 0.851±0.149 | 0.992±0.149 | ＜0.001 | 0.771 |  | 0.656 |  |
| 30 | 1.944±0.766 | 0.845 | 3.639 | 0.790±0.214 | 0.916±0.214 | ＜0.001 | 0.479 |  | 0.445 |  |

TableS19：Temporomandibular Joint L

The mean volumes (± SD) of the auto-contours of the Temporomandibular Joint-L for each patient, as well as the intersection and union volumes, sensitivity, specificity, the overall Kappa coefficient with its p-value (H₀: κ = 0), and the generalized conformity index (CI-gen) for each case. The overall Kappa coefficient with its p-value (H₀: κ = 0); p < 0.001 indicates agreement significantly higher than random.

| Case | volume | Intersection volume | Union volume | Sensitivity | Specificity | p-value: | Overall kappa: | Mean±SD | CI-gen | Mean±SD |
| --- | --- | --- | --- | --- | --- | --- | --- | --- | --- | --- |
| 1 | 2.454±0.421 | 0.690 | 4.566 | 0.747±0.225 | 0.933±0.225 | ＜0.001 | 0.452 | 0.446±0.070 | 0.411 | 0.399±0.058 |
| 2 | 1.262±0.641 | 0.289 | 2.786 | 0.698±0.219 | 0.950±0.219 | ＜0.001 | 0.395 |  | 0.315 |  |
| 3 | 2.669±0.622 | 1.216 | 4.980 | 0.879±0.139 | 0.913±0.139 | ＜0.001 | 0.517 |  | 0.446 |  |
| 4 | 1.682±0.484 | 0.764 | 3.349 | 0.826±0.202 | 0.925±0.202 | ＜0.001 | 0.491 |  | 0.428 |  |
| 5 | 2.251±0.909 | 0.643 | 4.257 | 0.635±0.283 | 0.941±0.283 | ＜0.001 | 0.371 |  | 0.359 |  |
| 6 | 2.529±0.370 | 1.109 | 4.131 | 0.808±0.221 | 0.896±0.221 | ＜0.001 | 0.512 |  | 0.496 |  |
| 7 | 2.412±0.472 | 0.991 | 4.323 | 0.854±0.218 | 0.881±0.218 | ＜0.001 | 0.474 |  | 0.448 |  |
| 8 | 1.533±0.692 | 0.646 | 3.445 | 0.798±0.170 | 0.917±0.170 | ＜0.001 | 0.410 |  | 0.354 |  |
| 9 | 2.080±0.433 | 0.876 | 3.701 | 0.794±0.201 | 0.918±0.201 | ＜0.001 | 0.506 |  | 0.450 |  |
| 10 | 3.757±1.032 | 0.825 | 7.032 | 0.673±0.264 | 0.938±0.264 | ＜0.001 | 0.398 |  | 0.370 |  |
| 11 | 1.907±0.364 | 0.765 | 3.546 | 0.815±0.243 | 0.942±0.243 | ＜0.001 | 0.526 |  | 0.453 |  |
| 12 | 1.976±0.621 | 0.848 | 3.674 | 0.751±0.165 | 0.940±0.165 | ＜0.001 | 0.502 |  | 0.435 |  |
| 13 | 2.676±0.718 | 1.096 | 4.769 | 0.725±0.150 | 0.935±0.150 | ＜0.001 | 0.484 |  | 0.444 |  |
| 14 | 2.658±0.345 | 0.925 | 4.445 | 0.772±0.183 | 0.942±0.183 | ＜0.001 | 0.521 |  | 0.480 |  |
| 15 | 1.452±0.355 | 0.302 | 3.082 | 0.673±0.196 | 0.944±0.196 | ＜0.001 | 0.382 |  | 0.322 |  |
| 16 | 2.794±0.559 | 0.664 | 5.413 | 0.661±0.228 | 0.940±0.228 | ＜0.001 | 0.391 |  | 0.354 |  |
| 17 | 3.987±1.042 | 1.577 | 7.922 | 0.785±0.168 | 0.980±0.168 | ＜0.001 | 0.551 |  | 0.410 |  |
| 18 | 1.639±0.568 | 0.000 | 3.470 | 0.634±0.476 | 0.889±0.476 | ＜0.001 | 0.229 |  | 0.249 |  |
| 19 | 2.054±0.645 | 0.708 | 4.047 | 0.685±0.184 | 0.919±0.184 | ＜0.001 | 0.394 |  | 0.371 |  |
| 20 | 2.293±0.471 | 0.741 | 3.969 | 0.715±0.171 | 0.938±0.171 | ＜0.001 | 0.466 |  | 0.436 |  |
| 21 | 1.453±0.202 | 0.312 | 2.935 | 0.677±0.229 | 0.936±0.229 | ＜0.001 | 0.386 |  | 0.343 |  |
| 22 | 2.244±0.456 | 0.944 | 3.878 | 0.725±0.208 | 0.957±0.208 | ＜0.001 | 0.528 |  | 0.461 |  |
| 23 | 2.057±0.248 | 0.788 | 4.043 | 0.805±0.237 | 0.902±0.237 | ＜0.001 | 0.440 |  | 0.393 |  |
| 24 | 1.890±0.283 | 0.784 | 3.651 | 0.786±0.206 | 0.910±0.206 | ＜0.001 | 0.460 |  | 0.405 |  |
| 25 | 2.080±0.291 | 0.685 | 3.922 | 0.775±0.273 | 0.904±0.273 | ＜0.001 | 0.434 |  | 0.394 |  |
| 26 | 1.910±0.640 | 0.669 | 3.996 | 0.724±0.215 | 0.909±0.215 | ＜0.001 | 0.385 |  | 0.354 |  |
| 27 | 1.313±0.371 | 0.569 | 2.469 | 0.746±0.249 | 0.930±0.249 | ＜0.001 | 0.473 |  | 0.432 |  |
| 28 | 2.385±0.722 | 0.429 | 4.966 | 0.632±0.148 | 0.938±0.148 | ＜0.001 | 0.358 |  | 0.309 |  |
| 29 | 1.913±0.389 | 0.870 | 3.512 | 0.782±0.219 | 0.943±0.219 | ＜0.001 | 0.531 |  | 0.458 |  |
| 30 | 1.632±0.323 | 0.451 | 3.017 | 0.724±0.226 | 0.919±0.226 | ＜0.001 | 0.424 |  | 0.399 |  |

TableS20：Temporomandibular Joint R

The mean volumes (± SD) of the auto-contours of the Temporomandibular Joint-R for each patient, as well as the intersection and union volumes, sensitivity, specificity, the overall Kappa coefficient with its p-value (H₀: κ = 0), and the generalized conformity index (CI-gen) for each case. The overall Kappa coefficient with its p-value (H₀: κ = 0); p < 0.001 indicates agreement significantly higher than random.

| Case | volume | Intersection volume | Union volume | Sensitivity | Specificity | p-value: | Overall kappa: | Mean±SD | CI-gen | Mean±SD |
| --- | --- | --- | --- | --- | --- | --- | --- | --- | --- | --- |
| 1 | 23.715±3.555 | 14.837 | 31.625 | 0.845±0.094 | 0.981±0.015 | ＜0.001 | 0.746 | 0.755±0.073 | 0.676 | 0.659±0.084 |
| 2 | 4.857±3.484 | 1.633 | 10.225 | 0.737±0.232 | 0.960±0.075 | ＜0.001 | 0.465 |  | 0.361 |  |
| 3 | 17.510±3.615 | 11.751 | 23.764 | 0.856±0.143 | 0.984±0.016 | ＜0.001 | 0.769 |  | 0.681 |  |
| 4 | 23.103±3.359 | 16.687 | 30.326 | 0.883±0.090 | 0.985±0.013 | ＜0.001 | 0.802 |  | 0.722 |  |
| 5 | 25.543±2.790 | 16.039 | 35.520 | 0.844±0.067 | 0.981±0.012 | ＜0.001 | 0.742 |  | 0.655 |  |
| 6 | 9.159±2.522 | 5.564 | 13.364 | 0.853±0.168 | 0.980±0.025 | ＜0.001 | 0.730 |  | 0.628 |  |
| 7 | 8.522±2.459 | 4.741 | 12.981 | 0.837±0.175 | 0.984±0.022 | ＜0.001 | 0.708 |  | 0.590 |  |
| 8 | 4.513±1.432 | 2.187 | 7.226 | 0.783±0.181 | 0.985±0.017 | ＜0.001 | 0.659 |  | 0.533 |  |
| 9 | 15.932±2.913 | 11.061 | 21.164 | 0.881±0.130 | 0.986±0.011 | ＜0.001 | 0.797 |  | 0.708 |  |
| 10 | 20.324±3.477 | 14.705 | 25.954 | 0.886±0.120 | 0.984±0.018 | ＜0.001 | 0.806 |  | 0.738 |  |
| 11 | 19.856±3.601 | 13.891 | 25.999 | 0.884±0.123 | 0.983±0.017 | ＜0.001 | 0.793 |  | 0.717 |  |
| 12 | 22.053±6.721 | 9.127 | 34.691 | 0.762±0.176 | 0.984±0.017 | ＜0.001 | 0.636 |  | 0.520 |  |
| 13 | 29.854±5.761 | 17.587 | 43.489 | 0.832±0.109 | 0.991±0.007 | ＜0.001 | 0.743 |  | 0.619 |  |
| 14 | 13.225±2.443 | 8.927 | 17.637 | 0.870±0.130 | 0.985±0.015 | ＜0.001 | 0.783 |  | 0.697 |  |
| 15 | 35.585±5.068 | 26.760 | 45.347 | 0.898±0.096 | 0.993±0.007 | ＜0.001 | 0.841 |  | 0.752 |  |
| 16 | 18.890±3.728 | 12.055 | 26.766 | 0.839±0.121 | 0.988±0.009 | ＜0.001 | 0.756 |  | 0.646 |  |
| 17 | 11.030±1.883 | 7.820 | 14.286 | 0.879±0.121 | 0.985±0.013 | ＜0.001 | 0.800 |  | 0.724 |  |
| 18 | 19.478±3.120 | 14.336 | 25.669 | 0.894±0.087 | 0.987±0.016 | ＜0.001 | 0.815 |  | 0.728 |  |
| 19 | 13.632±3.977 | 6.562 | 21.834 | 0.776±0.179 | 0.994±0.005 | ＜0.001 | 0.677 |  | 0.530 |  |
| 20 | 20.948±3.212 | 15.333 | 26.328 | 0.889±0.115 | 0.987±0.008 | ＜0.001 | 0.820 |  | 0.749 |  |
| 21 | 10.854±2.600 | 6.655 | 15.773 | 0.843±0.154 | 0.987±0.015 | ＜0.001 | 0.742 |  | 0.628 |  |
| 22 | 15.816±3.252 | 10.331 | 21.767 | 0.869±0.130 | 0.982±0.021 | ＜0.001 | 0.765 |  | 0.675 |  |
| 23 | 17.363±3.283 | 11.650 | 23.157 | 0.868±0.141 | 0.984±0.012 | ＜0.001 | 0.779 |  | 0.695 |  |
| 24 | 16.430±2.036 | 11.650 | 21.513 | 0.885±0.069 | 0.986±0.014 | ＜0.001 | 0.803 |  | 0.722 |  |
| 25 | 20.161±4.569 | 11.944 | 28.221 | 0.833±0.155 | 0.985±0.014 | ＜0.001 | 0.737 |  | 0.639 |  |
| 26 | 16.773±3.317 | 11.636 | 22.550 | 0.886±0.119 | 0.986±0.018 | ＜0.001 | 0.795 |  | 0.704 |  |
| 27 | 12.673±2.812 | 7.920 | 17.577 | 0.851±0.157 | 0.987±0.009 | ＜0.001 | 0.761 |  | 0.658 |  |
| 28 | 18.045±3.420 | 13.643 | 22.284 | 0.913±0.124 | 0.984±0.014 | ＜0.001 | 0.806 |  | 0.725 |  |
| 29 | 13.820±3.248 | 8.756 | 19.336 | 0.851±0.149 | 0.992±0.010 | ＜0.001 | 0.771 |  | 0.656 |  |
| 30 | 17.073±3.301 | 12.135 | 23.286 | 0.874±0.096 | 0.997±0.005 | ＜0.001 | 0.814 |  | 0.698 |  |

TableS21：Thyroid Gland

The mean volumes (± SD) of the auto-contours of the Thyroid Gland for each patient, as well as the intersection and union volumes, sensitivity, specificity, the overall Kappa coefficient with its p-value (H₀: κ = 0), and the generalized conformity index (CI-gen) for each case. The overall Kappa coefficient with its p-value (H₀: κ = 0); p < 0.001 indicates agreement significantly higher than random.

| Case | volume | Intersection volume | Union volume | Sensitivity | Specificity | p-value: | Overall kappa: | Mean±SD | CI-gen | Mean±SD |
| --- | --- | --- | --- | --- | --- | --- | --- | --- | --- | --- |
| 1 | 28.273±6.458 | 17.290 | 43.244 | 0.866±0.127 | 0.996±0.005 | ＜0.001 | 0.751 | 0.717±0.076 | 0.611 | 0.593±0.073 |
| 2 | 31.968±7.182 | 20.511 | 49.007 | 0.859±0.097 | 0.993±0.009 | ＜0.001 | 0.749 |  | 0.614 |  |
| 3 | 50.741±9.273 | 33.376 | 73.524 | 0.878±0.081 | 0.993±0.007 | ＜0.001 | 0.778 |  | 0.654 |  |
| 4 | 26.100±9.191 | 5.550 | 38.233 | 0.768±0.343 | 0.985±0.014 | ＜0.001 | 0.611 |  | 0.509 |  |
| 5 | 42.764±6.424 | 29.150 | 59.111 | 0.887±0.100 | 0.992±0.008 | ＜0.001 | 0.799 |  | 0.687 |  |
| 6 | 38.892±8.853 | 24.403 | 59.166 | 0.866±0.110 | 0.995±0.006 | ＜0.001 | 0.754 |  | 0.617 |  |
| 7 | 37.091±6.919 | 24.771 | 52.457 | 0.882±0.096 | 0.993±0.009 | ＜0.001 | 0.789 |  | 0.671 |  |
| 8 | 42.876±17.501 | 9.766 | 61.107 | 0.750±0.318 | 0.993±0.002 | ＜0.001 | 0.632 |  | 0.519 |  |
| 9 | 31.818±13.074 | 7.026 | 44.462 | 0.775±0.360 | 0.992±0.006 | ＜0.001 | 0.645 |  | 0.536 |  |
| 10 | 35.750±10.637 | 20.699 | 56.709 | 0.842±0.102 | 0.995±0.008 | ＜0.001 | 0.723 |  | 0.578 |  |
| 11 | 48.494±22.906 | 7.995 | 69.699 | 0.758±0.379 | 0.994±0.004 | ＜0.001 | 0.631 |  | 0.505 |  |
| 12 | 33.101±14.002 | 6.787 | 50.057 | 0.731±0.314 | 0.996±0.003 | ＜0.001 | 0.627 |  | 0.483 |  |
| 13 | 49.819±8.367 | 33.582 | 67.478 | 0.885±0.084 | 0.997±0.003 | ＜0.001 | 0.815 |  | 0.696 |  |
| 14 | 29.394±8.013 | 17.357 | 46.472 | 0.862±0.114 | 0.996±0.005 | ＜0.001 | 0.734 |  | 0.588 |  |
| 15 | 36.459±9.125 | 22.388 | 54.765 | 0.867±0.108 | 0.995±0.007 | ＜0.001 | 0.758 |  | 0.622 |  |
| 16 | 35.678±16.771 | 5.779 | 51.446 | 0.757±0.406 | 0.994±0.005 | ＜0.001 | 0.629 |  | 0.503 |  |
| 17 | 28.693±4.871 | 19.161 | 40.006 | 0.873±0.099 | 0.995±0.004 | ＜0.001 | 0.796 |  | 0.675 |  |
| 18 | 39.892±9.450 | 24.481 | 60.109 | 0.856±0.104 | 0.996±0.005 | ＜0.001 | 0.755 |  | 0.616 |  |
| 19 | 47.114±10.963 | 27.745 | 69.634 | 0.859±0.119 | 0.994±0.008 | ＜0.001 | 0.755 |  | 0.622 |  |
| 20 | 32.327±5.067 | 19.364 | 45.829 | 0.849±0.088 | 0.996±0.004 | ＜0.001 | 0.772 |  | 0.643 |  |
| 21 | 49.010±13.148 | 31.126 | 73.739 | 0.881±0.103 | 0.995±0.008 | ＜0.001 | 0.763 |  | 0.630 |  |
| 22 | 37.484±8.505 | 22.542 | 56.602 | 0.871±0.116 | 0.996±0.005 | ＜0.001 | 0.755 |  | 0.617 |  |
| 23 | 35.311±7.440 | 21.696 | 52.224 | 0.856±0.111 | 0.993±0.007 | ＜0.001 | 0.756 |  | 0.626 |  |
| 24 | 26.698±9.943 | 7.636 | 36.515 | 0.792±0.330 | 0.983±0.013 | ＜0.001 | 0.643 |  | 0.560 |  |
| 25 | 55.786±10.986 | 38.613 | 78.030 | 0.890±0.083 | 0.996±0.005 | ＜0.001 | 0.806 |  | 0.685 |  |
| 26 | 33.758±12.262 | 11.270 | 51.586 | 0.786±0.275 | 0.986±0.018 | ＜0.001 | 0.646 |  | 0.522 |  |
| 27 | 47.195±5.564 | 33.516 | 60.350 | 0.880±0.080 | 0.995±0.004 | ＜0.001 | 0.831 |  | 0.733 |  |
| 28 | 28.698±12.751 | 9.354 | 41.401 | 0.737±0.385 | 0.991±0.009 | ＜0.001 | 0.567 |  | 0.457 |  |
| 29 | 35.214±15.592 | 6.735 | 51.064 | 0.763±0.373 | 0.988±0.011 | ＜0.001 | 0.615 |  | 0.507 |  |
| 30 | 36.546±15.779 | 8.567 | 53.393 | 0.773±0.360 | 0.990±0.011 | ＜0.001 | 0.639 |  | 0.517 |  |

TableS22：Trachea

The mean volumes (± SD) of the auto-contours of the Trachea for each patient, as well as the intersection and union volumes, sensitivity, specificity, the overall Kappa coefficient with its p-value (H₀: κ = 0), and the generalized conformity index (CI-gen) for each case. The overall Kappa coefficient with its p-value (H₀: κ = 0); p < 0.001 indicates agreement significantly higher than random.

| Case | volume | Intersection volume | Union volume | Sensitivity | Specificity | p-value: | Overall kappa: | Mean±SD | CI-gen | Mean±SD |
| --- | --- | --- | --- | --- | --- | --- | --- | --- | --- | --- |
| 1 | 37.489±11.327 | 23.73 | 51.2456 | 0.810±0.186 | 0.998±0.001 | ＜0.001 | 0.630 | 0.588±0.067 | 0.46 | 0.505±0.066 |
| 2 | 51.235±11.226 | 38.45 | 64.0208 | 0.868±0.108 | 0.956±0.037 | ＜0.001 | 0.668 |  | 0.60 |  |
| 3 | 62.607±13.938 | 41.24 | 83.9729 | 0.813±0.107 | 0.976±0.015 | ＜0.001 | 0.616 |  | 0.49 |  |
| 4 | 54.775±11.887 | 37.39 | 72.1595 | 0.826±0.105 | 0.953±0.030 | ＜0.001 | 0.596 |  | 0.52 |  |
| 5 | 33.087±17.276 | 18.94 | 47.2356 | 0.776±0.260 | 0.956±0.052 | ＜0.001 | 0.488 |  | 0.40 |  |
| 6 | 31.644±4.481 | 22.49 | 40.7955 | 0.841±0.069 | 0.952±0.022 | ＜0.001 | 0.624 |  | 0.55 |  |
| 7 | 40.739±2.178 | 25.34 | 56.1396 | 0.784±0.025 | 0.962±0.005 | ＜0.001 | 0.556 |  | 0.45 |  |
| 8 | 24.545±6.370 | 17.05 | 32.0362 | 0.835±0.126 | 0.956±0.036 | ＜0.001 | 0.613 |  | 0.53 |  |
| 9 | 70.536±21.482 | 46.26 | 94.8122 | 0.813±0.143 | 0.960±0.035 | ＜0.001 | 0.582 |  | 0.49 |  |
| 10 | 55.166±2.590 | 38.72 | 71.6097 | 0.834±0.022 | 0.959±0.006 | ＜0.001 | 0.627 |  | 0.54 |  |
| 11 | 39.876±2.766 | 27.59 | 52.1641 | 0.826±0.034 | 0.940±0.012 | ＜0.001 | 0.587 |  | 0.53 |  |
| 12 | 48.755±12.838 | 32.60 | 64.9096 | 0.819±0.126 | 0.955±0.034 | ＜0.001 | 0.586 |  | 0.50 |  |
| 13 | 34.583±1.701 | 23.13 | 46.0386 | 0.815±0.023 | 0.971±0.004 | ＜0.001 | 0.618 |  | 0.50 |  |
| 14 | 32.981±3.891 | 21.98 | 43.9767 | 0.812±0.054 | 0.955±0.015 | ＜0.001 | 0.587 |  | 0.50 |  |
| 15 | 57.993±25.548 | 36.42 | 79.5659 | 0.805±0.216 | 0.961±0.046 | ＜0.001 | 0.553 |  | 0.46 |  |
| 16 | 32.509±0.224 | 20.64 | 44.3809 | 0.791±0.003 | 0.956±0.001 | ＜0.001 | 0.559 |  | 0.46 |  |
| 17 | 38.328±13.492 | 23.74 | 52.9169 | 0.793±0.167 | 0.963±0.033 | ＜0.001 | 0.551 |  | 0.45 |  |
| 18 | 53.018±2.362 | 41.19 | 64.8497 | 0.879±0.023 | 0.953±0.008 | ＜0.001 | 0.691 |  | 0.64 |  |
| 19 | 34.340±5.350 | 24.44 | 44.2374 | 0.843±0.080 | 0.966±0.016 | ＜0.001 | 0.650 |  | 0.55 |  |
| 20 | 56.998±13.600 | 37.13 | 76.8636 | 0.806±0.116 | 0.950±0.032 | ＜0.001 | 0.561 |  | 0.48 |  |
| 21 | 63.006±15.226 | 49.93 | 76.0775 | 0.894±0.119 | 0.976±0.028 | ＜0.001 | 0.746 |  | 0.66 |  |
| 22 | 41.269±0.895 | 25.90 | 56.6409 | 0.786±0.010 | 0.951±0.003 | ＜0.001 | 0.543 |  | 0.46 |  |
| 23 | 70.247±16.252 | 51.50 | 88.9926 | 0.858±0.118 | 0.961±0.031 | ＜0.001 | 0.660 |  | 0.58 |  |
| 24 | 73.474±34.633 | 43.24 | 103.7036 | 0.780±0.231 | 0.924±0.083 | ＜0.001 | 0.446 |  | 0.42 |  |
| 25 | 36.939±22.363 | 20.54 | 53.3423 | 0.775±0.303 | 0.953±0.064 | ＜0.001 | 0.462 |  | 0.38 |  |
| 26 | 22.568±1.347 | 13.92 | 31.2168 | 0.777±0.028 | 0.942±0.008 | ＜0.001 | 0.517 |  | 0.45 |  |
| 27 | 30.589±7.208 | 20.23 | 40.9524 | 0.814±0.111 | 0.964±0.024 | ＜0.001 | 0.596 |  | 0.49 |  |
| 28 | 31.857±0.528 | 22.35 | 41.364 | 0.833±0.008 | 0.951±0.003 | ＜0.001 | 0.614 |  | 0.54 |  |
| 29 | 24.347±10.238 | 15.67 | 33.0287 | 0.814±0.209 | 0.966±0.039 | ＜0.001 | 0.578 |  | 0.47 |  |
| 30 | 34.161±16.460 | 20.76 | 47.5569 | 0.795±0.238 | 0.941±0.070 | ＜0.001 | 0.496 |  | 0.44 |  |

TableS23：GTV

The mean volumes (± SD) of the auto-contours of the GTV for each patient, as well as the intersection and union volumes, sensitivity, specificity, the overall Kappa coefficient with its p-value (H₀: κ = 0), and the generalized conformity index (CI-gen) for each case. The overall Kappa coefficient with its p-value (H₀: κ = 0); p < 0.001 indicates agreement significantly higher than random.
